# Dynamic Alterations of Extracellular Polymeric Substances and Their Associations with Microbial Communities in the Soil Plastisphere

**DOI:** 10.3390/microorganisms14030546

**Published:** 2026-02-27

**Authors:** Wenjuan Liu, Wenjuan Zhai, Xiufeng Wan, Jiahe Wang, Yongfei Ren, Wenbo Deng

**Affiliations:** 1 Observation and Research Station of the Ministry of Education of Shanxi Subalpine Grassland Ecosystem, Institute of Loess Plateau, Shanxi University, Taiyuan 030006, China; 2 College of Environment and Resource, Shanxi University, Taiyuan 030006, China; 3 China Institute for Radiation Protection, Taiyuan 030006, China

**Keywords:** plastisphere, extracellular polymeric substances, biofilm, microbiomes, soil ecosystems

## Abstract

Extracellular polymeric substances (EPS) facilitate microbiome adhesion on microplastic surfaces and ensure matrix cohesion, playing a crucial role in establishing the structure and function of the plastisphere. Nevertheless, the dynamic alterations in the composition and features of plastisphere EPS and their relationships with biotic and abiotic factors remain poorly understood, especially in soil ecosystems. The study investigated the variations in the EPS secretion behavior of the plastisphere using three types of microplastics across three representative soils with three incubation durations. Results showed that plastisphere EPS had a more complex composition and lower aromaticity, apparent molecular weight, and polarity than natural soil dissolved organic matter did. Continuous changes in EPS composition and features were detected during incubation. The bacterial plastisphere community played a central role in regulating EPS secretion, and other factors (such as soil properties, incubation time and microplastic types) influenced EPS secretion via the bacterial composition of the plastisphere. A decrease in the number of microbial OTUs was significantly correlated with EPS components that governed the dynamics of the EPS composition and features of the plastisphere during incubation, a pattern that was particularly evident for bacteriomes. This study advances our insight into microbiome-EPS interactions within the soil plastisphere and deepens our understanding of its formation mechanisms.

## 1. Introduction

The increasing consumption of plastic products, combined with improper use and disposal strategies, has caused widespread accumulation of microplastics (MPs, <5 mm in diameter) globally [1,2]. MP contamination has become a central concern in ecological and environmental research and is recognized as a factor that influences global climate change [3,4]. Additionally, MPs can increase the bioavailability of co-pollutants (e.g., heavy metals) and their associated ecotoxicity to microbes and plants [5]. Once they enter the natural environment, MPs can act as a stable substrate for microbial colonization and growth, which causes the formation of biofilms and thereby creates novel anthropogenic ecological niches on MPs (i.e., the plastisphere) [6,7]. Soil ecosystems now constitute the principal reservoir of MPs, representing an estimated 52% of the global inventory because of extensive plastic discharge [8,9]. Therefore, the soil plastisphere has become a hotspot for microbiomes, and substantial plastisphere biomass is present in global soils [10]. Furthermore, the plastisphere has been demonstrated to strongly influence ecological functions in soil environments, such as element cycling and the degradation of xenobiotic substances [11]. Despite the crucial role of the plastisphere in soil ecosystems, terrestrial research lags behind aquatic studies, and our understanding of the dynamic alterations and factors influencing soil plastispheres is still limited [12].

The composition of the plastisphere includes microbiomes (e.g., bacteria and fungi) and extracellular polymeric substances (EPS) [13,14,15]. Data on the plastisphere remain scarce; however, it is well established that in biofilms, EPS typically constitute up to 80% of the dry mass and 90% of the total organic content per unit volume [16]. The EPS matrix comprises proteins, polysaccharides and humic substances, and is commonly used in water treatment, colour removal from wastewater, and the removal of toxic organic compounds. For example, synthesised EPS exhibits good flocculating performance and industrial potential for drinking water treatment, achieving maximum chemical oxygen demand and turbidity removal rates of 61.2% and 95.6%, respectively [17]. Additionally, EPS provides a framework that supports the mechanical stability of biofilms and promotes the adhesion of microbiomes to the surface of microplastics [18,19]. These hydrated biomolecules not only provide many supportive functions for the microbiomes within the plastispheres but also influence the interactions between pollutants and MPs [20]. Given the importance of EPS, exploring the composition and features of EPS on MPs is essential for understanding the formation and succession of the plastisphere.

Previous studies on the plastisphere have focused mainly on microbial communities [21,22,23]; however, interest has recently shifted towards exploring the composition of EPS on MP surfaces. Liu et al. [24] reported that EPS accumulated on the surface of polypropylene incubated in an aquatic environment, with humic acids and proteins being the main components. A recent study investigated the composition of EPS within the aquatic plastisphere by determining protein, polysaccharide and humic substance contents and exploring their relationships with the MPs-associated bacteriome [25]. Despite recent progress in EPS research within the aquatic plastisphere, EPS secretion dynamics during microbial colonization of MPs in soil ecosystems remain poorly understood. Additionally, other factors in addition to the bacterial plastisphere community may affect the EPS compositional profiles associated with MPs. Fungi, which have been found to colonize and grow on MPs in ecosystems, also produce EPS [26]. For example, certain *Candida* spp. have been shown to produce EPS that contribute to biofilm formation [27]. Furthermore, EPS secreted by fungi have attracted increasing interest because of their strong viscosity and hydrophilicity, which enable them to increase soil water retention and provide structural support during the formation of microaggregates [26]. In addition to microbial community effects, environmental properties could influence the composition of EPS [28,29]. To date, limited studies have investigated how these factors modulate EPS within the plastisphere, particularly in soil ecosystems. Addressing this knowledge gap is the key concern of the present study.

We performed a 90-day soil microcosm experiment using three representative soils, i.e., red, black and yellow-brown soils, with different properties and three MPs, i.e., polyethylene (PE), polyamide (PA), and polylactic acid (PLA), with distinct physicochemical properties that are ubiquitous in soil ecosystems [30,31,32]. The present study aimed to (1) decipher the dynamic alterations in the composition and features of EPS within plastispheres over time across various soils; (2) elucidate the effects of biotic (bacterial and fungal plastisphere communities) and abiotic (soil properties and MP types) factors on the EPS of the plastisphere; and (3) explore the process and mechanism of EPS secretion in the soil plastisphere. The present study elucidated EPS secretion dynamics during soil plastisphere formation and explored their relationships with MP-associated microbiomes and soil properties.

## 2. Materials and Methods

### 2.1. Soil Collection and MP Preparation

Three soils (red, black and yellow-brown soils) were chosen for this research on the basis of their distinct soil characteristics and climate zones. Topsoils were sampled from the 0–20 cm layer of red, black and yellow-brown soils in Guangxi (21.35° N, 110.82° E), Jilin (41.68° N, 129.12° E), and Shanxi (38.63° N, 113.87° E) Provinces in China, respectively. All soils were obtained from forested areas, with sampling sites located far from human habitation and minimally affected by MP pollution [33]. After being air-dried at ambient temperature, the soil samples were passed through a 2 mm mesh for subsequent microcosm experiments.

Three types of MPs (PE, PA and PLA) were obtained from Xinmiao New Material Co., Ltd. (Dongguan, Guangzhou, China). All the MPs had a diameter of approximately 100 μm, corresponding to the predominant size range (20–200 μm) of the MPs found in soil environments [34,35]. The MPs underwent a comprehensive cleaning procedure using ethanol and ultrapure water and were subsequently air-dried at ambient temperature, and then preserved at 4 °C until use. The properties of the MPs and soil samples were determined, and the relevant results were depicted in our recent study [33].

### 2.2. Plastisphere Incubation Experimental Design

The mesh bag method was employed to investigate the EPS and microbiomes of the plastisphere because nearly 100 μm-sized MPs are difficult to collect directly from soil [36]. This method is frequently used to study the composition of microbiomes within plastispheres incubated in aquatic and soil environments [19,36,37]. Prior to the plastisphere incubation, all the air-dried soil samples were preincubated at 75% water-holding capacity and 25 °C for two weeks to reactivate the endogenous soil microbiomes. The plastisphere incubation experimental setup was described in our recent study [33]. Briefly, after thorough disinfection, 1 g of MP particles was placed into each mesh bag (80 μm mesh), and the bags were buried in different soil treatments. For soil plastisphere incubation, the mesh bags with MPs were incubated in glass containers (20 cm long, 14 cm wide and 16 cm high) containing 1.5 kg of soil (dry weight). All the treatments were repeated four times. During MP biofilm formation, all glass containers were sealed with breathable membranes and maintained at 25 °C in complete darkness within an incubator. Sterilized water was added every three days to keep the soil moisture constant for each treatment during the entire incubation process. Three durations, i.e., 30, 60, 90 days, were used in the present study. The mesh bags were destructively collected on each sampling day. A total of 108 plastisphere samples were collected in the present study, and the samples, which were cleaned with ultrapure water three times, were divided into two parts: one was employed to characterize the EPS composition and features of the plastisphere, and the other was employed to analyze the microbiomes within the plastisphere. Additionally, corresponding soil samples were collected on each sampling day, after which the soil properties were determined. In terms of notation/sample identification, the PE MP sample incubated in yellow-brown soil and sampled after 90 days of incubation is called “YPE90”, and so forth.

### 2.3. Sample Characterization

The properties of the soil samples, such as pH, soil organic carbon (SOC), available P (AP) and N (i.e., NH_4_^+^-N and NO_3_^−^-N) and dissolved organic carbon (DOC), were measured on the basis of established protocols [33]. The results of the soil property measurements are shown in Figure S1. The accumulation of EPS on the surface of the MPs was detected by field emission scanning electron microscopy (FESEM; Merlin Compact, Zeiss, Oberkochen, Germany). Before the FESEM analysis, all the samples were coated with 25 nm of platinum. The bacterial and fungal communities within the plastispheres were analyzed by 16S rRNA and ITS high-throughput sequencing, respectively. Details of the plastisphere microbiome analysis are provided in Supplementary Materials.

Prior to EPS composition analysis, EPS were extracted from the plastisphere samples in aqueous solution. The extraction was conducted according to methods described in previous studies [24,38]. Briefly, 100 mg of each plastisphere sample was added to a beaker that contained 2.0 mL of NaOH (0.01 M) and sonicated for 1 h at 100 W and 40 kHz. Afterwards, the pH of the solution was adjusted to 7 using 0.01 M HCl. The supernatant was used to analyze the composition and features of EPS within the plastispheres.

EPS have been regarded as “the dark matter of biofilm” because of the large mass of matrix biopolymers within them, which is similar to dissolved organic matter (DOM) in the natural environment [16,39]. Conventional chemical analysis may not fully characterize the overall composition and functional features of EPS. Spectral methods, including ultraviolet-visible (UV-Vis) and three-dimensional excitation-emission matrix (3D-EEM) fluorescence, which were typically used to characterize natural DOM [40,41], have been applied to analyze the features and composition of plastisphere EPS [11,19]. The UV-Vis absorption spectra of EPS were determined at wavelengths of 200–700 nm by a UV-Vis spectrometer (Cary 300, Varian, Palo Alto, CA, USA). The calculation methods for the spectral parameters, including SUVA254, SUVA280 and E_253_/E_203_, are detailed in Supplementary Materials. The 3D-EEM fluorescence spectra of soil DOM and plastisphere EPS were determined by a fluorescence spectrophotometer (F2710, Hitachi, Tokyo, Japan). The compositional information of the soil DOM and plastisphere EPS was analyzed by parallel factor analysis (PARAFAC) modeling using the R package “staRdom”. The details of the fluorescence determination, as well as the calculation methods for the fluorescence parameters, including the biological index (BIX), fluorescence index (FI), humification index (HIX) and freshness index, are depicted in Supplementary Materials. The content of DOC, which is a vital indicator of EPS concentration [11], in plastisphere EPS was determined by a Multi N/C 2100S TOC analyzer (Analytikjena, Jena, Germany).

### 2.4. Statistical Analysis

The relative abundances of main EPS components and the values of key EPS parameters are shown in Tables S1 and S2, respectively. Statistical differences in parameters across treatments were determined by one-way analysis of variance followed by Duncan’s multiple range test (*p* < 0.05). Permutation multivariate analysis of variance (PERMANOVA) was employed to assess the effects of the incubation environment (soil properties), exposure duration and plastic type on EPS composition derived from PARAFAC modeling. The temporal turnover rates of EPS composition were calculated from the slope of linear least-squares regression conducted between compositional dissimilarity (1-Bray–Curtis similarity) and incubation time intervals. These calculations were performed using the R (4.5.0) package “vegan”.

Hierarchical partitioning was performed by the R package “rdacca.hp” to assess the relative contributions of soil properties and microbial plastisphere communities to EPS composition, with individual factor effects isolated through variance partitioning [42]. Random forest analyses of which microbial taxa were the main predictors driving the alterations in individual EPS components were conducted with the R (4.5.0) package “randomForest”. Relationships between influencing factors (soil properties, diversity and composition of microbial plastisphere communities) and EPS composition were assessed using a Mantel test (R package “linkET”), with the composition of microbial plastisphere communities represented by the first two principal coordinates (PCoA1 and PCoA2) from PCoA.

Partial least squares path modeling (PLS-PM) with the R (4.5.0) package “plspm” was employed to analyze the direct and indirect pathways of factors affecting the EPS composition of the plastisphere. To examine relationship dynamics, interaction networks between plastisphere microbial OTUs and individual EPS components were constructed across incubation durations, with microbial OTUs classified into topological modules. A module represents a cluster of microbial taxa that are strongly connected within a relevant function. Network construction was based on relationships with Spearman correlation coefficients ≥ 0.4 (*p* < 0.05), and visualization was conducted using Cytoscape 3.9.1.

## 3. Results and Discussion

### 3.1. The Accumulation of EPS on MP Surface

**Figure 1:**
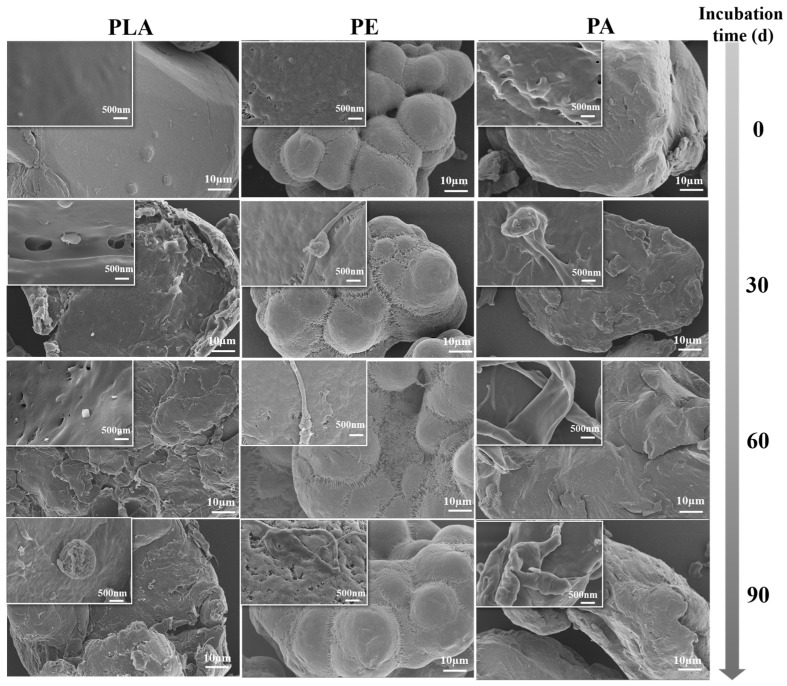
Field emission scanning electron microscopy images of three types of microplastics before and after incubation in red soil.

As conducive substrates for microbial colonization and proliferation, MPs can stimulate EPS secretion and enhance biofilm formation [43]. FESEM images revealed the accumulation of EPS on the surfaces of MPs incubated in different soils throughout the entire incubation period (Figure 1 and Figures S2 and S3). The surfaces of all the MPs were covered by biofilms after 30 days of incubation, and the EPS thickness increased progressively with incubation time. The accumulation of EPS caused the surfaces of the MPs to become rough, and compared with the other two MPs, more EPS accumulated on the surface of PA. The DOC contents of EPS extracted from various MPs incubated in different soils increased gradually throughout the incubation period, indicating the continual accumulation of EPS on the surface of the MPs (Figure S4). Consistent with our findings, a previous study demonstrated that microbiome colonization on MP surfaces occurred after a few days of incubation in seawater, followed by sustained EPS secretion during biofilm development [44]. The DOC contents of EPS extracted from PA were clearly higher (*p* < 0.05) than those extracted from the other MPs after 90 days of incubation. While the DOC content of EPS extracted from PLA was significantly higher than that from PE incubated in yellow-brown soil, the inverse was true in black soil. Additionally, the EPS accumulation rates, derived from the rates of DOC change within EPS, showed that EPS accumulated more rapidly during the early and middle stages of incubation than in the late stage (Table S3). Moreover, the accumulation rates on PA were consistently higher than those on PLA and PE throughout the incubation period. These results demonstrated that plastic type, incubation time and the colonization environment (soil properties) could influence the accumulation of EPS on the MP surface.

### 3.2. Dynamic Alterations of Components and Features of Plastisphere EPS

Spectroscopic analysis was used to determine the components and features of the plastisphere EPS. SUVA254 and SUVA280, derived from UV-Vis spectroscopic analysis, were calculated to characterize the aromatization and apparent molecular weight of the plastisphere EPS [45,46,47,48]. The two parameters showed different alteration trends across the soils; however, both were commonly higher in the early stage of incubation than in the late stage (Figure S5). This possibly indicated that some labile-like compounds accumulated in EPS during incubation. The E_253_/E_203_ ratio characterized the number of substituent groups, such as hydroxyl and carbonyl groups, on the benzene ring of the plastisphere EPS and was positively related to the electron transfer capacity of the EPS [49,50]. The E_253_/E_203_ ratio of EPS extracted from various MPs incubated in different soils tended to increase overall during incubation. These findings were consistent with the results of Fourier transform infrared analysis in our recent study, which demonstrated that the abundance of oxygen-containing groups within the plastisphere increased significantly with incubation time [33]. Energy-dispersive spectroscopy analysis revealed that the oxygen content of aquatic plastisphere samples increased after incubation [24]. The authors attributed this increase to the continuous formation of oxygen-containing groups, which is consistent with the findings of the present study [24]. Additionally, the increased electron transfer capacity of plastisphere EPS could provide more electrons for microbial metabolism, thereby promoting the colonization of surrounding microbes on MP surfaces [44]. The *t*-test results revealed that the SUVA254, SUVA280, and E_253_/E_203_ values of the plastisphere EPS were all significantly lower (*p* < 0.01) than those of the soil DOM (Figure S6), suggesting that the EPS had less aromaticity, apparent molecular weight, and polarity compared to soil DOMs.

**Figure 2:**
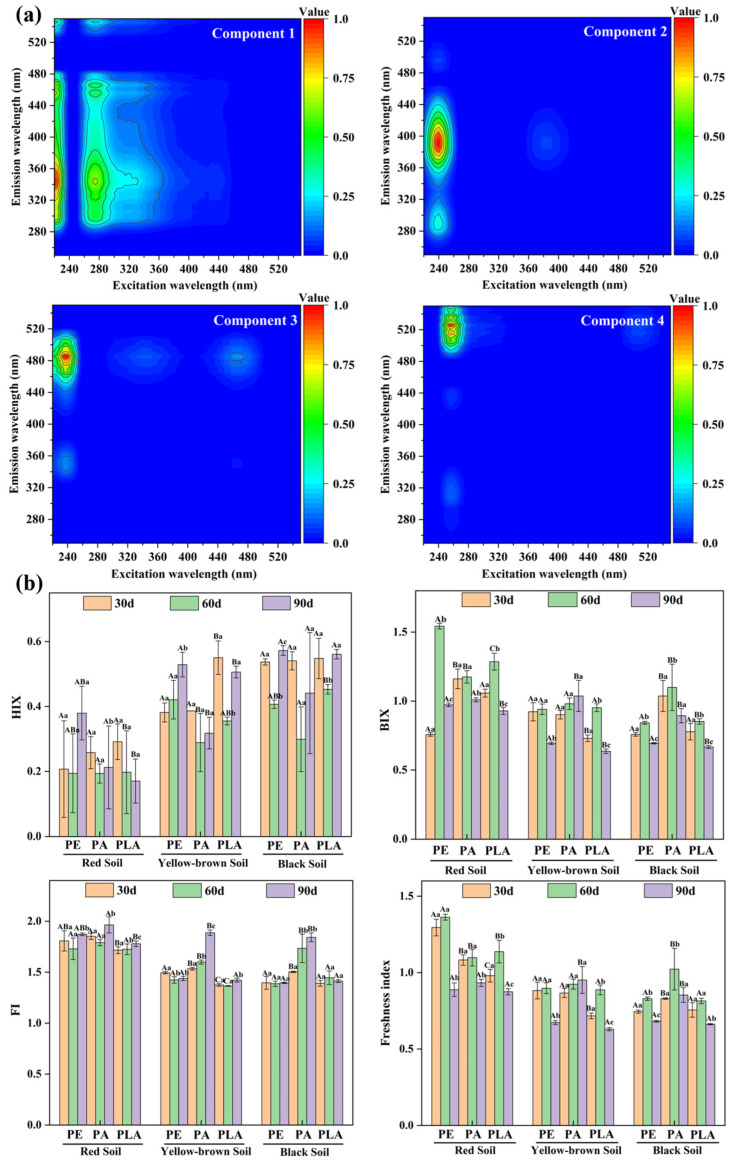
Composition and features of plastisphere extracellular polymeric substances. (**a**) Four main components of plastisphere extracellular polymeric substances derived from parallel factor analysis modeling; (**b**) Temporal changes in the fluorescence parameters of plastisphere extracellular polymeric substances. The capital letters indicate significant differences in the fluorescence parameters of extracellular polymeric substances from different MPs incubated in the same soil for the same incubation duration, while the lowercase letters indicate significant differences in the fluorescence parameters of extracellular polymeric substances from the same MPs incubated in the same soil across different incubation durations. The significance threshold was set at 0.05.

The two-component and four-component models were validated by Tucker’s congruence coefficients (>0.95; Tables S4 and S5) for soil DOMs and plastisphere EPS, respectively. According to the OpenFluor database (similarity > 0.95), the two components for soil DOM were assigned to a terrestrial humic-like component [51,52] and a mixture of humic-like and tryptophan-like substances [53], respectively (Figure S7). In contrast, the composition of the plastisphere EPS was more complex (Figure 2a). Component 1 showed a maximum intensity at 220/350 nm (excitation/emission) and was assigned as a tryptophan-like protein substance on the basis of the OpenFluor database (similarity > 0.90) [54]. Component 2 showed a maximum intensity at 240/390 nm (excitation/emission) and was assigned as a tyrosine-like protein substance based on the OpenFluor database (similarity > 0.95) [55]. Fluorescence components 3 and 4 for the plastisphere EPS, which had maximum intensities at 237/480 and 255/520 nm (excitation/emission), were absent from the OpenFluor database. According to the traditional classification method, components 3 and 4 were assigned as fulvic acid-like substances and humic acid-like substances [56], respectively, and both components belong to humic substances.

Conventionally, humic substances within the biofilm were regarded as derived from the surrounding natural DOM [57]. Additionally, derivatives from MP degradation (such as low-molecular-weight oligomers and DOM) that contain humic-like substances may accumulate in EPS [58,59]. Both humic substances were absent from the OpenFluor database, which might indicate that both components are a mixture of microbial EPS (labile compounds) and humic substances, resulting in them being less complex and less recalcitrant than natural humic substances [60]. This situation demonstrates that plastisphere EPS is an intricately complex mixture of recalcitrant and labile organic substances, such that high-resolution techniques like Fourier transform ion cyclotron resonance mass spectrometry (FT-ICR-MS) should be used to obtain a comprehensive molecular-level profile of EPS in the future.

Fluorescence indices were used to determine the changes in EPS features during incubation. The values of HIX, which characterize the humification of EPS [61,62], typically tend to initially decrease but then increase, especially for EPS extracted from MPs incubated in black soil. Both the BIX and freshness index values, which are positively correlated with the content of microbiome-sourced EPS, typically demonstrated a trend of an initial increase followed by a decrease during incubation. However, the mechanism driving the changes in EPS features in the plastisphere during incubation, particularly the relationship between the microbiome and EPS composition, requires further study. FI is typically employed to distinguish EPS sourced from terrestrial origins (FI < 1.4) or microbial activity (FI > 1.7) [40]. The FI values for EPS extracted from red soil were greater than those for EPS extracted from yellow-brown and black soil. This was attributed to the relatively low DOC content in red soil (Figure S1), leading to less terrestrial DOM being absorbed onto the plastisphere compared with that in the other two soils. EPS in the plastisphere was generally verified to be a mixture of natural DOM and organic substances derived from microbial metabolism, as indicated by the FI values (1.4 < FI < 1.7) [40].

The relative abundance of EPS component 1 generally decreased during the incubation. In contrast, EPS component 2 was relatively abundant on the surfaces of PA and PLA in red soil, as well as on PE in yellow-brown and black soil, after 60 days of incubation (Figure S8). It was difficult to summarize any consistent pattern from the changes in components 3 and 4 for EPS. This may be attributed to the complex composition of the two mixtures. PERMANOVA has been frequently used to study the composition of microbiomes and has been recently applied to natural DOM [63]. In the present study, PERMANOVA indicated that the colonization environment, incubation time, and plastic type all exerted a significant influence (*p* < 0.05) on the EPS composition of the plastisphere (Figure S9). Pathway analysis needs to be employed to clarify how these factors affect EPS composition.

To clarify the temporal variation in EPS composition from different MPs, we calculated the time decay of EPS dissimilarity [40]. The results verified that incubation time once again had a significant influence (*p* < 0.05) on EPS composition (Figure S10). The temporal turnover rate of EPS extracted from PA was the highest among the three MPs, followed by that extracted from PLA and PE. This phenomenon is possibly attributed to the greater microbial biomass on the PA surface than on the other MPs [33], which resulted in higher microbial activity and, consequently, a higher EPS turnover rate.

### 3.3. Drivers of EPS Secretion in the Plastisphere

**Figure 3:**
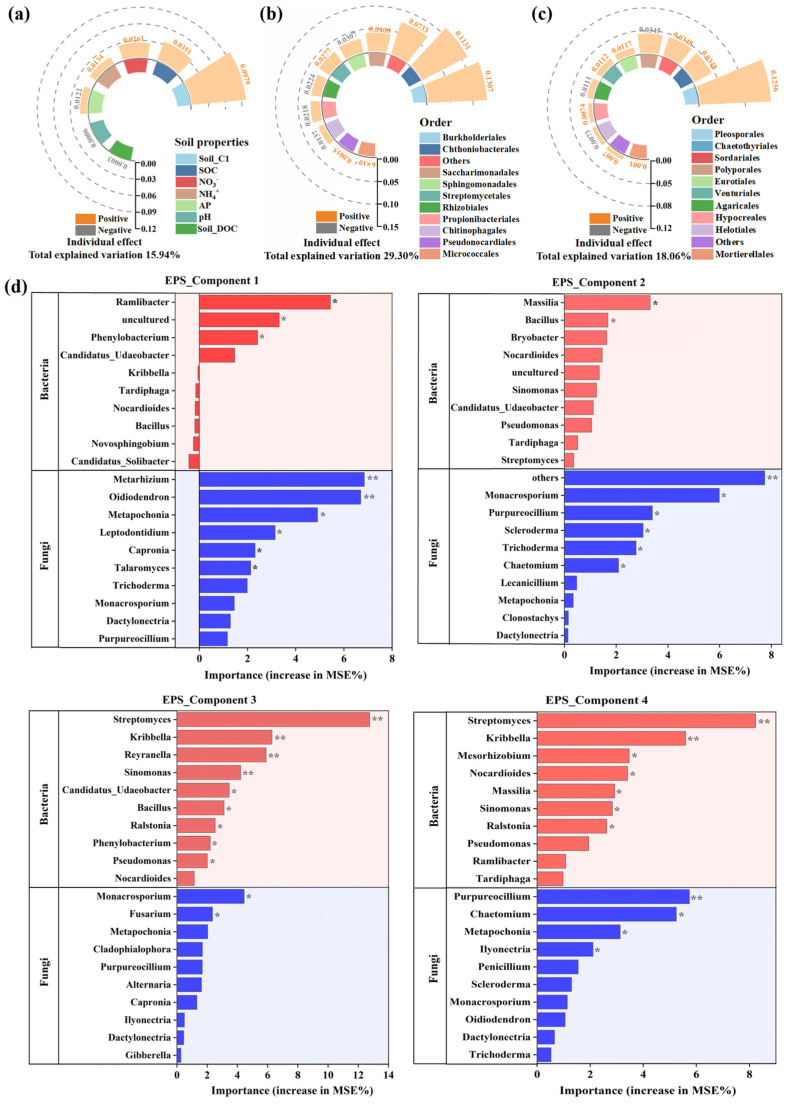
Contributions of soil properties and plastisphere microbial communities (order-level, top 10 taxa) to extracellular polymeric substance secretion derived from partitioning analysis: (**a**) soil property parameters, (**b**) bacterial community, and (**c**) fungal community. (**d**) Random forest analysis was employed to identify the most important microbial plastisphere genera (bacterial and fungal) influencing individual extracellular polymeric substance components. Models were constructed using the top 30 taxa by abundance, and the top 10 predictors for bacterial and fungal communities are displayed in the figure. Significance levels: * *p* < 0.05, ** *p* < 0.01.

The compositions of the bacterial and fungal plastisphere communities at the order and genus levels during incubation are shown in Figures S11 and S12. Compared with the bulk soil, a greater proportion of the plastisphere microbiome was concentrated within the top 10 most abundant taxa for both bacteria and fungi. This finding indicates that a less diverse but unique microbial community assembled within the plastisphere across all three soils during incubation. Hierarchical partitioning based on canonical correspondence analysis revealed that the bacterial plastisphere community (29.30%) played the most important role in EPS composition during incubation, followed by the fungal plastisphere (18.06%) community and soil properties (15.94%) (Figure 3a–c). Most members in the three groups positively influenced EPS composition variation. The Orders Burkholderiales (0.13) and Chthoniobacterales (0.11) emerged as the major contributors to plastisphere EPS composition in the bacterial community, demonstrating their important roles in EPS regulation within the plastisphere. Although microbial taxa from the orders Bacillales, Pseudomonadales, and Paenibacillales are commonly important contributors to soil biofilms [64], their relatively low abundance in the soil plastispheres means that they are not major contributors to plastisphere EPS. For example, Bacillales ranked 28th in terms of relative abundance among all bacterial taxa at the order level in the plastisphere bacteriome, resulting in a relatively minor effect on EPS composition. Burkholderiales have been frequently reported to accumulate significantly on the surfaces of MPs and are considered an important MP degrader [11,20]. The Family Comamonadaceae, which belongs to the order Burkholderiales, includes taxa reported to produce EPS in the soil plastisphere, indicating that Burkholderiales comprises both MP degraders and EPS producers [65]. Chthoniobacterales has rarely been reported as an EPS producer; however, it showed a strong positive relationship with components 3 (r^2^ = 0.58, *p* < 0.01) and 4 (r^2^ = 0.49, *p* < 0.01) of plastisphere EPS in the present study. The order Chthoniobacterales belongs to the phylum Verrucomicrobiota, which is widely distributed but difficult to culture [66]. A recent study verified that some taxa in this phylum can synthesize a variety of polysaccharides, and that their EPS-producing capacity is significantly enhanced in nitrogen-deficient environments [67]. This may explain why Chthoniobacterales played an important role in the composition of EPS on MPs, which constitute a severely nitrogen-deficient habitat.

The effect of the fungal community on EPS composition was obviously weaker than that of the plastisphere bacterial community. This is attributed to the difference in size between bacteria (0.5–5 μm) and fungi (2–10 μm) [68], which results in higher bacterial abundance and diversity on the MP surface (~100 μm) and, hence, a weaker fungal influence on EPS composition. Order Pleosporales was the major contributor to the plastisphere EPS composition in the fungal community. Previous studies have reported that some taxa belonging to Pleosporales, such as *Curvularia* and *Alternaria* [69,70], can produce large amounts of EPS. These EPS compounds have potential applications in the pharmaceutical industry due to their significant antioxidant, free radical scavenging, anticancer, and other biological activities [69,70]. Environmental properties were verified to have an important influence on EPS production, especially available nutrient elements (such as NH_4_^+^-N, NO_3_^−^-N, and AP) [15]. However, the effects of these factors were not as significant as those of the plastisphere microbiome. This is attributed to the limited access of microbiomes within the plastisphere to surrounding soil resources. Because they are encapsulated by EPS, plastisphere microbiomes obtain more nutrients from the EPS matrix and MPs than from the surrounding environment, particularly during the middle and late incubation stages [20,22]. The available soil nutrients did not play a significant role in influencing plastisphere EPS. Among the soil properties, the composition of soil DOM played the most important role in influencing EPS composition, verifying that plastisphere EPS contained natural DOM.

Correlation analysis was conducted to examine the relationships between dominant microbial genera (top 10 in abundance) and EPS components. The results showed that *Streptomyces* (bacteria) and *Purpureocillium* (fungi) exhibited consistently strong and statistically significant correlations (*p* < 0.05) with multiple EPS components (Table S6). Furthermore, Random Forest analysis indicated that the variation in EPS components C1–C4 could be partially explained by both bacterial and fungal taxa (top 30 in abundance) at the genus level. However, compared with bacterial taxa, fungal taxa explained a greater proportion of the variation in EPS components 1 and 2; conversely, bacterial taxa were more important in explaining the variation in components 3 and 4 (Figure 3d). This phenomenon demonstrated that EPS components 3 and 4 contained a significant amount of microbial metabolites, especially bacterial metabolites, and confirmed the above speculation. Many bacterial taxa, including *Bacillus*, *Phenylobacterium*, *Pseudomonas*, *Massilia*, and *Streptomyces*, which are frequently reported as EPS producers, were strongly associated with EPS components in the present study [71,72,73]. For example, *Streptomyces*, belong to the phylum Actinobacteriota, an important EPS producer in the soil, has attracted widespread interest because its EPS possesses bioactivities such as antioxidant effects [72]. Some species in *Massilia* were reported to have exopolysaccharide synthesis capacity for biofilm adhesion [71]. To date, the study of fungal EPS producers has lagged behind that of bacteria. However, *Fusarium* and *Trichoderma*, which were strongly associated with EPS components in the present study, have been reported to be important fungal EPS producers [29].

### 3.4. Relationships Between Plastisphere EPS Composition and Influencing Factors

**Figure 4:**
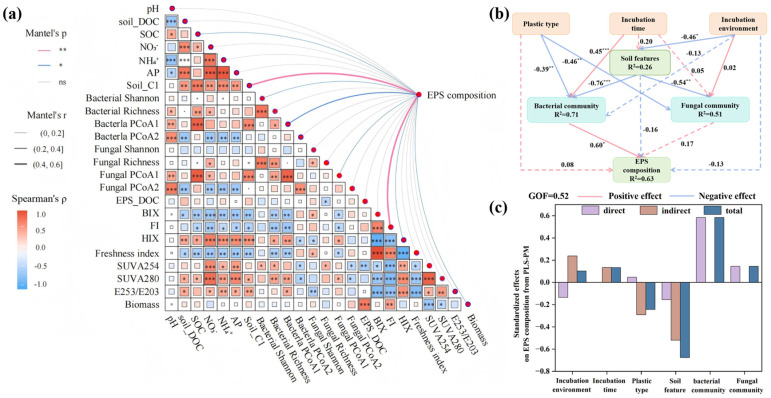
Relationships between the plastisphere extracellular polymeric substances and their influencing factors. (**a**) Mantel test-based relationships between EPS secretion and biotic/abiotic factors. Mantel’s r values were characterized by the edge width, and the significant correlations were characterized by the line colour. Pairwise Pearson coefficients between parameters were defined with a colour gradient. Levels of statistical significance are denoted as follows: * *p* < 0.05, ** *p* < 0.01; ns (not significant) denotes a lack of significant relationship between variables. The direct and indirect influences of biotic/abiotic factors on platisphere extracellular polymeric substances composition were evaluated by partial least squares path modeling (**b**,**c**). * for *p* < 0.05, ** for *p* < 0.01, and *** for *p* < 0.001.

The Mantel test was employed to assess the effects of soil properties and microbial communities on EPS composition. Soil DOM composition and SOC were significantly related to the composition of plastisphere EPS; additionally, other factors significantly related to EPS composition (such as FI, biomass, and bacterial PCoA1) were associated with the plastisphere microbiomes (Figure 4a). These findings suggested that microbiomes played a crucial role in the composition of plastisphere EPS. There is an inevitable exchange of nutrients and microorganisms between the plastisphere and the surrounding soil environment; however, the encapsulation and protection provided by EPS results in the formation of a highly self-sufficient, unique microenvironment within the plastisphere in soil ecosystems [6,22]. The soil plastisphere has been reported as a hot spot for antibiotic resistance genes [37], organic carbon degradation [33], and potential pathogens [74]. Given the unique structure and function of the plastisphere, more in-depth studies should be conducted to explore its impact on soil functions.

PLS-PM analysis was performed to elucidate the pathways through which biotic and abiotic factors influenced the composition of plastisphere EPS. The results revealed that abiotic factors, including the colonization environment, incubation time, plastic type, and soil properties, affected EPS composition by influencing the plastisphere microbiome, particularly the bacteriome (Figure 4b). For example, soil properties (total effect = −0.68) were the most important factor influencing EPS composition. However, most of this effect was indirect, operating through the plastisphere bacteriomes (indirect effect = −0.52; Figure 4c). Additionally, although PERMANOVA revealed that the colonization environment, incubation time, and plastic type significantly affected EPS composition, their direct effects were obviously weaker than their indirect effects, which were mediated by the plastisphere bacterial community. Therefore, many factors could influence the plastisphere EPS composition; nevertheless, the bacterial community was central to these influences and played the most important role in regulating EPS composition.

**Figure 5:**
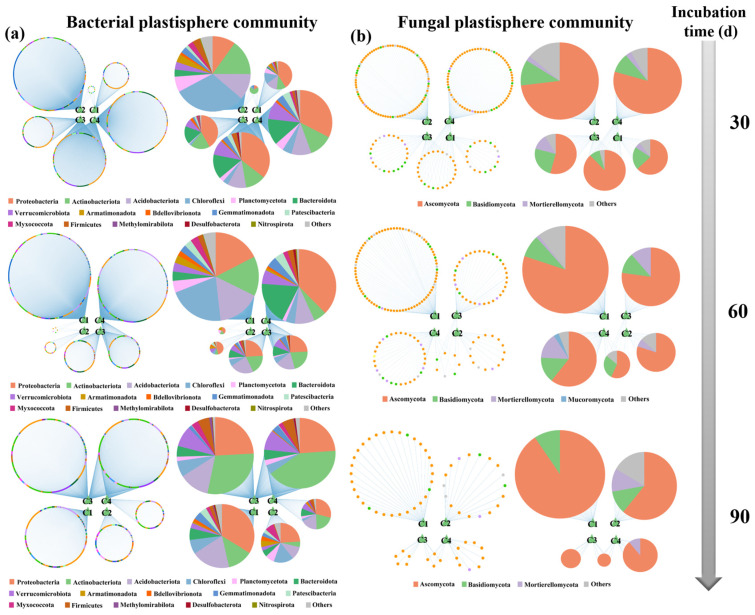
Networks of components of plastisphere extracellular polymeric substances and (**a**) bacterial OTUs and (**b**) fungal OTUs during the whole incubation process. The four main components of plastisphere extracellular polymeric substances, derived from parallel factor analysis modeling, are represented by C1 to C4.

To explore the dynamic changes in the relationships between microbiomes and EPS in the soil plastisphere, we constructed networks between microbial OTUs and EPS components at different incubation durations. As shown in Figure 5a,b, more members of the plastisphere bacteriome were strongly correlated with EPS components than those of the fungal community were, indicating that the bacterial plastisphere community plays a more crucial role in EPS secretion. Most fungal taxa related to EPS components belonged to the phyla Ascomycota and Basidiomycota, whereas bacterial taxa related to EPS components were distributed across many phyla, such as Proteobacteria, Actinobacteriota, and Acidobacteriota. Taxa belonging to these three phyla are frequently reported to be correlated with EPS production [25,75], and the first two (Proteobacteria and Actinobacteriota) are regarded as the main MP degraders in the plastisphere [33,76]. Common bacterial EPS producers were shared between components 1 and 2, and between components 3 and 4; however, there were almost no common EPS producers between the two groups, suggesting that the two groups had distinct sources. Additionally, the number of microbial OTUs in the networks decreased over the incubation period in both the bacterial and fungal communities (Tables S7 and S8). The number of bacterial OTUs correlated with components 1 and 2 was compared to that of OTUs correlated with components 3 and 4 early in the incubation process; nevertheless, the number of bacterial OTUs correlated with components 3 and 4 was significantly greater than that correlated with components 1 and 2 during the later stage of incubation. An inverse pattern was observed for fungal OTUs.

### 3.5. Process and Mechanism of Soil Plastisphere EPS Secretion

**Figure 6:**
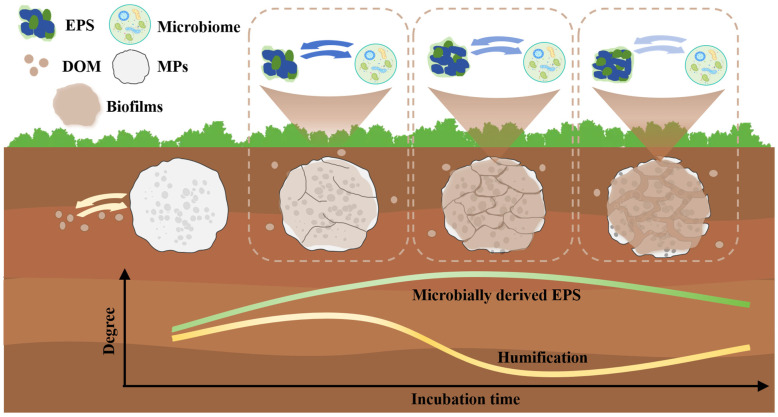
Conceptual diagram depicting the mechanisms and processes underlying extracellular polymeric substance secretion in the soil plastisphere.

The dynamic changes in the relationships between microbial OTUs and EPS components were regulated by the formation and succession of the soil plastisphere (Figure 6). Although it is difficult for microbes to colonize sterile MPs because of the scarcity of bioavailable nutrients, their greater hydrophobicity relative to soil particles facilitates the absorption of soil DOM, which can promote some pioneer species to colonize the MPs during the early incubation phase [22,77]. This phenomenon resulted in relatively high HIX values and a high summed abundance of EPS components 3 and 4 at this stage of incubation (Figure S13). As the incubation progressed, an increasing number of microbes that could utilize natural DOM (absorbed by the plastisphere) or MPs as nutrients colonized the MP surfaces and released EPS [22,33]. This activity not only resulted in decreased HIX values and increased BIX and freshness index values, but also led to a reduction in the summed abundance of EPS components 3 and 4 in the middle stage of incubation. Afterwards, many MP derivatives, such as low-molecular-weight oligomers and DOM, continued to be released with MP degradation, causing microbial toxicity and eliminating many pioneers that could not adapt to these substances [58,78]. This resulted in an obviously decrease in the number of bacterial OTUs related to EPS components (particularly components 1 and 2) at the biofilm maturation stage. Additionally, more than 50% of the bacterial OTUs associated with EPS components 3 and 4 belonged to the phyla Proteobacteria and Actinobacteriota, which have been reported to carry genes for MP degradation and EPS production, after 90 days of incubation [20,65]. Therefore, at this stage, the new EPS was derived mainly from extracellular enzymes for MP degradation and derivatives (containing many humic substances) from MP degradation. Additionally, some natural soil DOM was absorbed onto the plastispheres because their surface became rougher with incubation [79]. These factors could increase the values of HIX and the summed abundance of EPS components 3 and 4. The decrease in the values of the BIX and freshness index during this stage might be attributed to two reasons. First, the total number of microbial OTUs (especially bacterial OTUs) that significantly correlated with EPS components clearly decreased from the middle to the late stage of incubation. Second, functions such as MP degradation and EPS production in core microbial taxa within the plastisphere may be diluted during the biofilm maturation stage [80].

While this study provides valuable insights into EPS secretion within the soil plastisphere, certain limitations should be acknowledged. Owing to resolution limitations, the composition of plastisphere EPS could not be revealed at the molecular level. Future work should employ advanced mass spectrometry (e.g., FT-ICR-MS) and proteomics to analyze EPS composition at the molecular level. Additionally, metagenomic sequencing should be employed not only to explore the functional potential of plastisphere microbiomes but also to combine these results with the molecular composition of EPS to investigate the underlying mechanisms of plastisphere EPS production.

## 4. Conclusions

EPS play an important role in supporting the structure and function of the plastisphere; however, studies on EPS have lagged behind those on microbiomes within the plastisphere, especially in soil ecosystems. To our knowledge, the present study is the first to explore the dynamics of EPS composition and establish links between EPS components and microbiomes within the plastisphere in soils. The composition and features of EPS continued to change during the incubation period, and the bacterial plastisphere community played a crucial role in regulating the composition of EPS. Future work should investigate how temporal changes in EPS composition and properties within MP biofilms influence their interactions with coexisting pollutants—a factor overlooked in previous plastisphere studies [81,82] despite evidence that biofilms enhance pollutant adsorption compared to pure MPs [15]. Additionally, the plastisphere has been regarded as a reservoir for the accumulation of pathogenic microorganisms [33]. The protective role of EPS may be an important factor contributing to the enrichment of pathogens on the plastisphere [16]. Therefore, investigating the relationship between EPS components and pathogenic microorganisms within the plastisphere is also a critical topic for future research.

## Data Availability

The original contributions presented in the study are included in the article/Supplementary Materials; further inquiries can be directed to the corresponding authors.

## Supplementary Materials

The following supporting information can be downloaded at: https://www.mdpi.com/article/10.3390/microorganisms14030546/s1, Text S1. DNA Extraction and High-Throughput Sequencing; Text S2. The calculation for ultraviolet-visible spectral (UV-Vis) parameters; Text S3. Processing of three-dimensional excitation emission matrix (3D-EEM); Figure S1. The properties of soil samples collected from different incubation durations (30, 60, 90 days). Figure S2. Field emission scanning electron microscopy images of three types of microplastics before and after incubation in black soil; Figure S3. Field emission scanning electron microscopy images of three types of microplastics before and after incubation in yellow-brown soil; Figure S4. Temporal changes in the contents of dissolved organic carbon in EPS during incubation; Figure S5. Temporal changes in UV-Vis spectral parameters of EPS, including SUVA254, SUVA280 and E_253_/E_203_, during incubation; Figure S6. Temporal changes in UV-Vis spectral parameters of soil DOM, including SUVA254, SUVA280 and E_253_/E_203_, during incubation; Figure S7. Two main components of soil DOM derived from parallel factor analysis modeling; Figure S8. The changes in the relative abundances of four main components of plastisphere EPS during the incubation period; Figure S9. PERMANOVA analysis was performed to measure the influence of colonization environment (a), polymer types (b) and incubation time (c) on the composition of plastisphere EPS; Figure S10. Response of similarity of plastisphere EPS composition to the temporal differences in incubation; Figure S11. Heatmap of the abundance of the top 10 most abundant (a) bacterial and (b) fungal orders in the plastispheres during the whole incubation; Figure S12. Heatmap of the abundance of the top 10 most abundant (a) bacterial and (b) fungal genera in the plastisphere during the entire incubation period; Figure S13. Relative abundance of the sums of EPS components 1 + 2 and components 3 + 4; Table S1. Mean and SD values of EPS components; Table S2. Mean and SD values of DOC and key EPS parameters; Table S3. The accumulation rate of dissolved organic carbon (DOC) in EPS; Table S4. Results of Tucker’s Congruency Coefficients (TCC) for soil dissolved organic matter; Table S5. Results of Tucker’s Congruency Coefficients (TCC) for plastisphere extracellular polymeric substances; Table S6. Spearman correlation coefficients between the top 10 bacterial and fungal genera and EPS components; Table S7. Bacterial OTUs serving as nodes in the networks between components of plastisphere extracellular polymeric substances and bacterial community; Table S8. Fungal OTUs serve as nodes in the networks between components of plastisphere extracellular polymeric substances and the fungal community. References [11,40,41,45,61,62,83] are cited in the Supplementary Materials.
