# Supplementary material for: Dynamic Alterations of Extracellular Polymeric Substances and Their Associations with Microbial Communities in the Soil Plastisphere
Source: Microorganisms. 2026 Feb 27;14(3):546. doi: 10.3390/microorganisms14030546 (PMC13029585; doi:10.3390/microorganisms14030546)
Supplement: Supplementary file 1 [file microorganisms-14-00546-s001.zip › microorganisms-4122601-supplementary.pdf]

Dynamic Alterations of Extracellular Polymeric Substances and Their Associations with Microbial Communities in the Soil Plastisphere

Wenjuan Liu [1], Wenjuan Zhai [1], Xiufeng Wan [2], Jiahe Wang [2], Yongfei Ren [3] and Wenbo Deng [1,*]

[1] *Observation and Research Station of the Ministry of Education of Shanxi Subalpine Grassland Ecosystem, Institute of Loess Plateau, Shanxi University, Taiyuan 030006, China; ; liuwenjuan@sxu.edu.cn (W.L.); 202323202003@email.sxu.edu.cn (W.Z.)*

[2] *College of Environment and Resource, Shanxi University, Taiyuan 030006, China; 202301402139@email.sxu.edu.cn (X.W.); 20231402142@email.sxu.edu.cn (J.W.)*

[3] *China Institute for Radiation Protection, Taiyuan 030006, China; renyongfei0603@163.com*

* Correspondence: dengwb@sxu.edu.cn; Tel./Fax: +86-0351-7010700

This supplementary material includes:

3 Texts, 13 Figures, and 8 Tables

Text S1. DNA Extraction and High-Throughput Sequencing.

Text S2. The calculation for ultraviolet-visible spectral (UV-Vis) parameters.

Text S3. Processing of three-dimensional excitation emission matrix (3D-EEM).

Figure S1. The properties of soil samples collected from different incubation durations (30, 60, 90 days). The capital letters indicate significant differences in soil property across different incubation durations within same soil type, while the lowercase letters indicate significant differences in soil property among different soil types at the same incubation time. The significance threshold was set at 0.05.

Figure S2. Field emission scanning electron microscopy images of three types of microplastics before and after incubation in black soil.

Figure S3. Field emission scanning electron microscopy images of three types of microplastics before and after incubation in yellow-brown soil.

Figure S4. Temporal changes in the contents of dissolved organic carbon in EPS during incubation. The capital letters indicate significant differences of the contents of dissolved organic carbon in EPS from different MPs incubated in the same soil for same incubation duration, while the lowercase letters indicate significant differences of the contents of dissolved organic carbon in EPS from the same MPs incubated in the same soil across different incubation durations. The significance threshold was set at 0.05.

Figure S5. Temporal changes in UV-Vis spectral parameters of EPS, including SUVA254, SUVA 280 and E_{253}/E_{203}, during incubation. The capital letters indicate the significant differences of the spectral parameters of EPS from different MPs incubated in the same soil for same incubation duration, while the lowercase letters indicate significant differences in spectral parameters of EPS from the same MPs incubated in the same soil across different incubation durations. The significance threshold was set at 0.05.

Figure S6. Temporal changes in UV-Vis spectral parameters of soil DOM, including SUVA254, SUVA280 and E_{253}/E_{203}, during incubation. The capital letters indicate significant differences in the UV-Vis spectral parameters among different soil types at

the same incubation time, while the lowercase letters indicate significant differences in UV-Vis spectral parameters across different incubation durations within same soil type. The significance threshold was set at 0.05.

Figure S7. Two main components of soil DOM derived from parallel factor analysis modeling.

Figure S8. The changes of relative abundances of four main components of plastisphere EPS during incubation period. The capital letters indicate significant differences in the relative abundances of EPS components from different MPs incubated in the same soil for same incubation duration, while the lowercase letters indicate significant differences in the relative abundances in EPS components from the same MPs incubated in the same soil across different incubation durations. The significance threshold was set at 0.05.

Figure S9. PEARMANOVA analysis was performed to measure the influence of colonization environment (a), polymer types (b) and incubation time (c) on the composition of plastisphere EPS.

Figure S10. Response of similarity of plastisphere EPS composition to the temporal differences in incubation. * represent p <0.05, ** represent p <0.01, *** represent p <0.001.

Figure S11. Heatmap of the abundance of the top 10 most abundant (a) bacterial and (b) fungal orders in the plastispheres during the whole incubation.

Figure S12. Heatmap of the abundance of the top 10 most abundant (a) bacterial and (b) fungal genera in the plastisphere during the entire incubation period.

Figure S13. Relative abundance of the sums of EPS components 1+2 and components 3+4. The four main components of plastisphere extracellular polymeric substances, derived from parallel factor analysis, are designated C1 to C4.

Table S1. Mean and SD values of EPS components

Table S2. Mean and SD values of DOC and key EPS parameters

Table S3. The accumulation rate of dissolved organic carbon (DOC) in EPS

Table S4. Results of Tucker's Congruency Coefficients (TCC) for soil dissolved organic matter. Commonly, TCC > 0.95 indicates a good similarity.

Table S5. Results of Tucker's Congruency Coefficients (TCC) for plastisphere extracellular polymeric substances. Commonly, TCC > 0.95 indicates a good similarity.

Table S6. Spearman correlation coefficients between top 10 bacterial and fungal genera and EPS components.

Table S7. Bacterial OTUs serving as nodes in the networks between components of plastisphere extracellular polymeric substances and bacterial community. The four main components of plastisphere extracellular polymeric substances, derived from parallel factor analysis modeling, were represented by C1 to C4.

Table S8. Fungal OTUs serving as nodes in the networks between components of plastisphere extracellular polymeric substances and fungal community. The four main components of plastisphere extracellular polymeric substances, derived from parallel factor analysis modeling, were represented by C1 to C4.

Text S1. DNA Extraction and High-Throughput Sequencing.

The microbial community genomic DNA was extracted from soil and plastisphere samples using the ALFA-SEQ Magnetic Soil DNA Kit. DNA extraction quality was assessed by 1.5% agarose gel electrophoresis, while DNA concentration and purity were determined using a NanoDrop One spectrophotometer. The V3-V4 hypervariable region of the 16S rRNA gene was amplified and sequenced using primers 338F (ACTCCTACGGGAGGCAGCA) and 806R (GGACTACHVGGGTWTCTAAT) for bacterial community analysis. The ITS1-2 region of the fungal internal transcribed spacer (ITS) was amplified using primers BD-ITS1F (CTTGGTCATTTAGAGGAAGAAGTAA) and ITS2-2043R (GCTGCGTTCTTCATCGATGC) for fungal community analysis. Sequencing of the amplicon DNA libraries was performed on the Illumina NovaSeq 6000 platform by Guangdong Magigene Biotechnology Co., Ltd. Raw sequencing reads were quality-filtered using Fastp. Operational taxonomic units (OTUs) were assembled using usearch-fastq_mergepairs, and clustered using the unoise3 algorithm at 97% similarity threshold, with chimeric sequences removed. Taxonomic annotation of representative OTU sequences was performed against the bacterial 16S Silva v138 database and the fungal ITS Unite v8.0 database,

with a confidence threshold of 0.8. Sequencing data have been uploaded to the NCBI Sequence Read Archive database under BioProject accession number PRJNA1222479.

Text S2. The calculation for ultraviolet-visible spectral (UV-Vis) parameters

The absorbance spectra of plastisphere extracellular polymeric substances were measured at wavelength of 200-700 nm using Cary 300 UV-Vis spectrometer (Varian, USA), using pure water as reference solution. SUVA254 and SUVA280 were calculated by dividing the ultraviolet absorbances of plastisphere EPS at 254 nm and 280 nm by the dissolved organic carbon (DOC) concentration of corresponding EPS, respectively [1]. The spectral ratios E_{253}/E_{203} were obtained from the corresponding absorbance ratios (253 nm : 203 nm) [1].

Text S3. Processing of three-dimensional excitation emission matrix (3D-EEM).

The 3D-EEMs of soil dissolved organic matter (DOM) and plastisphere EPS samples were determined with a fluorescence spectrophotometer (F-2710, Hitachi, Japan) and applying pure water as blank. The scans ranged from 220-550 nm and 250-550 nm for excitation (Ex) and emission (Em), respectively, which with scan interval of 5 nm and 2 nm for Ex and Em, respectively. Before analysis, in order to eliminating inner-filter effect, the soil DOM samples and plastisphere EPS samples were diluted until the absorbance at 254 nm was lower than 0.3. The main components of soil DOM were identified with parallel factor analysis (PARAFAC) modeling applying the “staRdom” package in the R-computing environment. The results of PARAFAC were validated by the Tucker’s congruency coefficients (>0.95), and the maximum fluorescence intensity of the peak of the determined components was applied to calculate the relative proportions of the various components of soil DOM and plastisphere EPS samples [2-4].

Biological index (BIX), fluorescence index (FI), humification index (HIX), and freshness index were obtained as below: BIX, is defined as the ratio of the fluorescence intensity at the emission wavelength of 380 nm

and 430 nm when the excitation wavelength is 310 nm, which was usually applied to reveal the source of DOM [5]. The higher value of BIX indicated higher contribution of microbial activity to DOM [4]. FI, is calculated as the ratio of the fluorescence intensity at the emission wavelength of 470 nm and 520 nm when the excitation wavelength is 370 nm (as below formula). FI is usually applied to distinguish DOM sourced from terrestrial organisms (FI < 1.4) or microbial activity (FI > 1.7) [6]. HIX, is calculated as the ration of the integral of the fluorescence intensity at the emission wavelength of 435-480 nm and 300-345 nm when the excitation wavelength is 254nm. The HIX is an indictor to characterize the humification of DOM [7]. When the excitation wavelength is set to 310 nm, the ratio of the fluorescence intensity at the emission wavelength of 380 nm to the maximum fluorescence intensity within the range of 420-435 nm represents the freshness index [5]. This index was positively correlated with the DOM sourced from microbial communities [5].

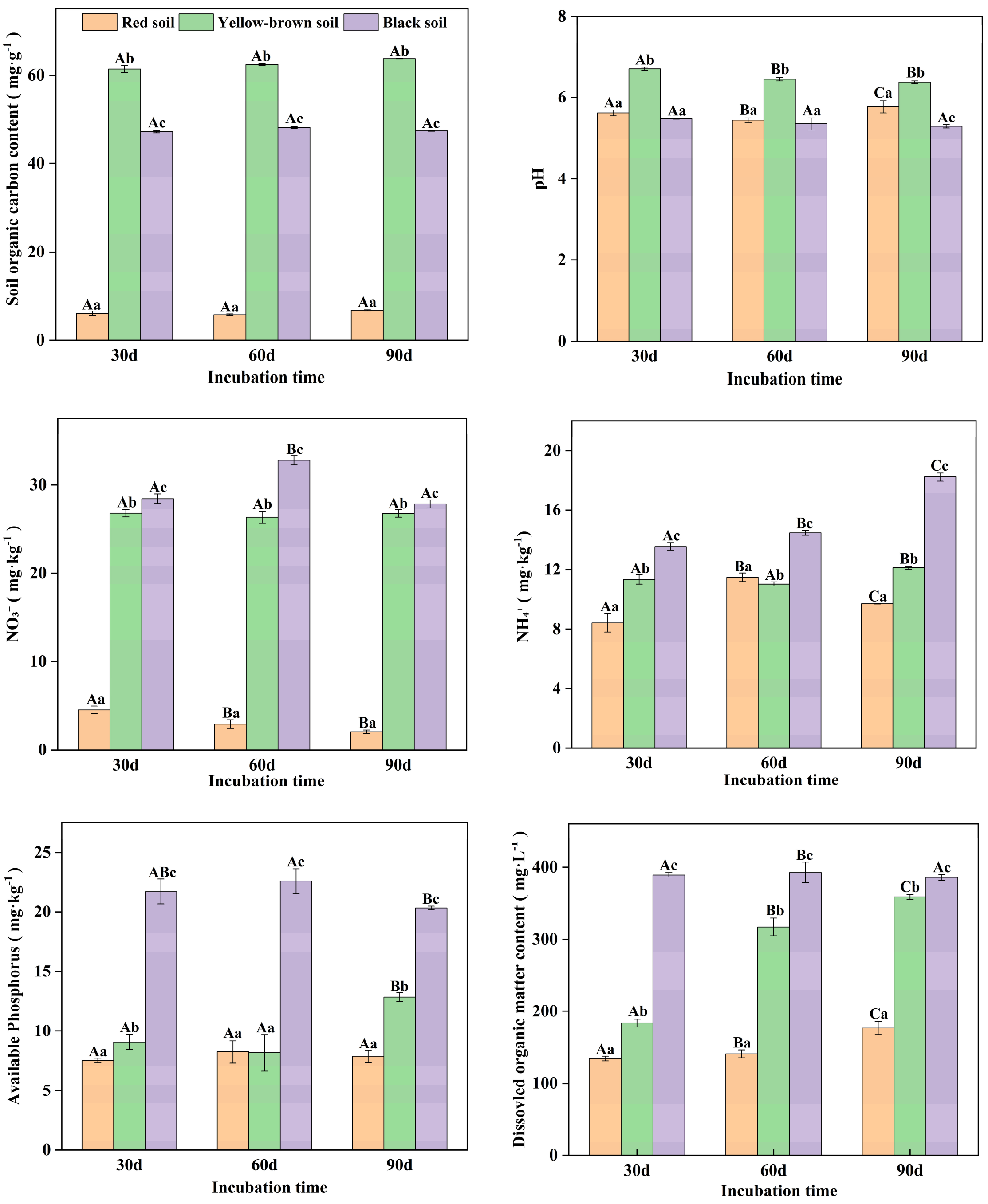

Figure S1. The properties of soil samples collected from different incubation durations (30, 60, 90 days). The capital letters indicate significant differences in soil property across different incubation durations within same soil type, while the lowercase letters indicate significant differences in soil property among different soil types at the same incubation time. The significance threshold was set at 0.05.

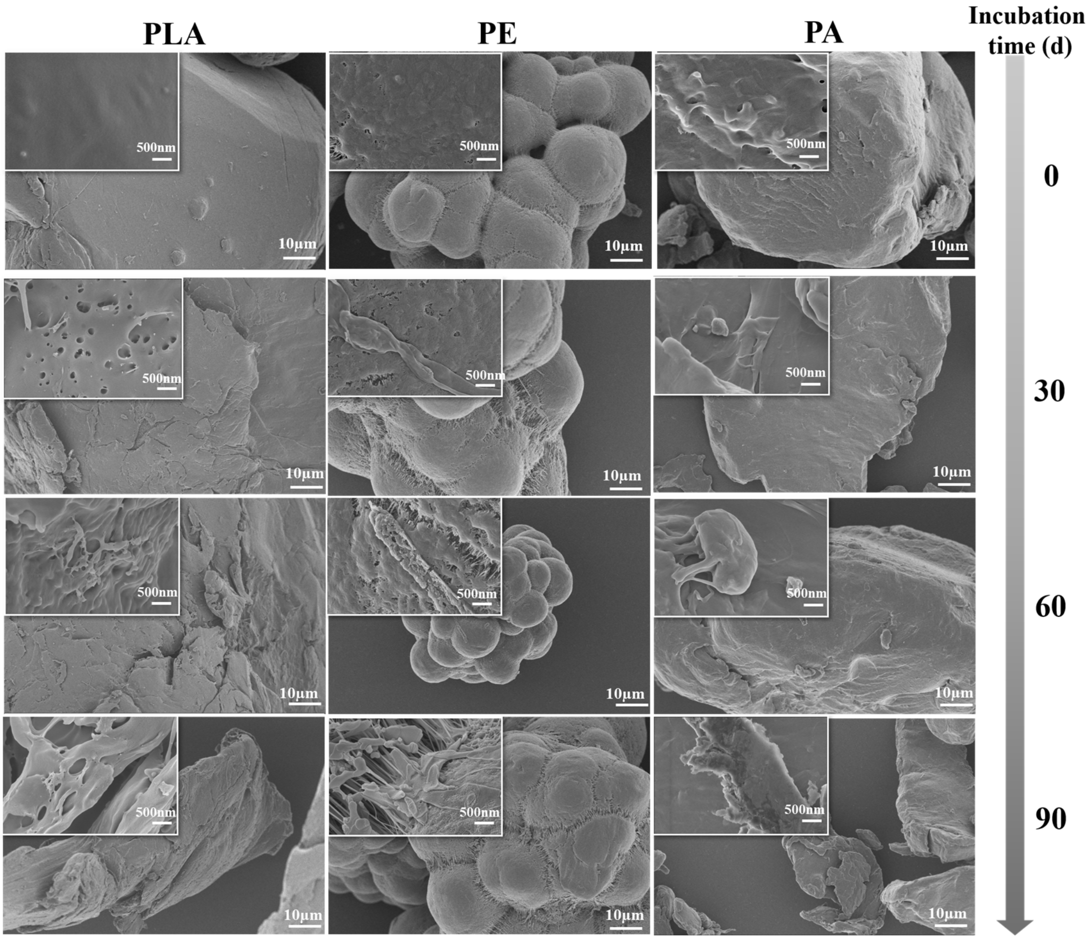

Figure S2. Field emission scanning electron microscopy images of three types of microplastics before and after incubation in black soil.

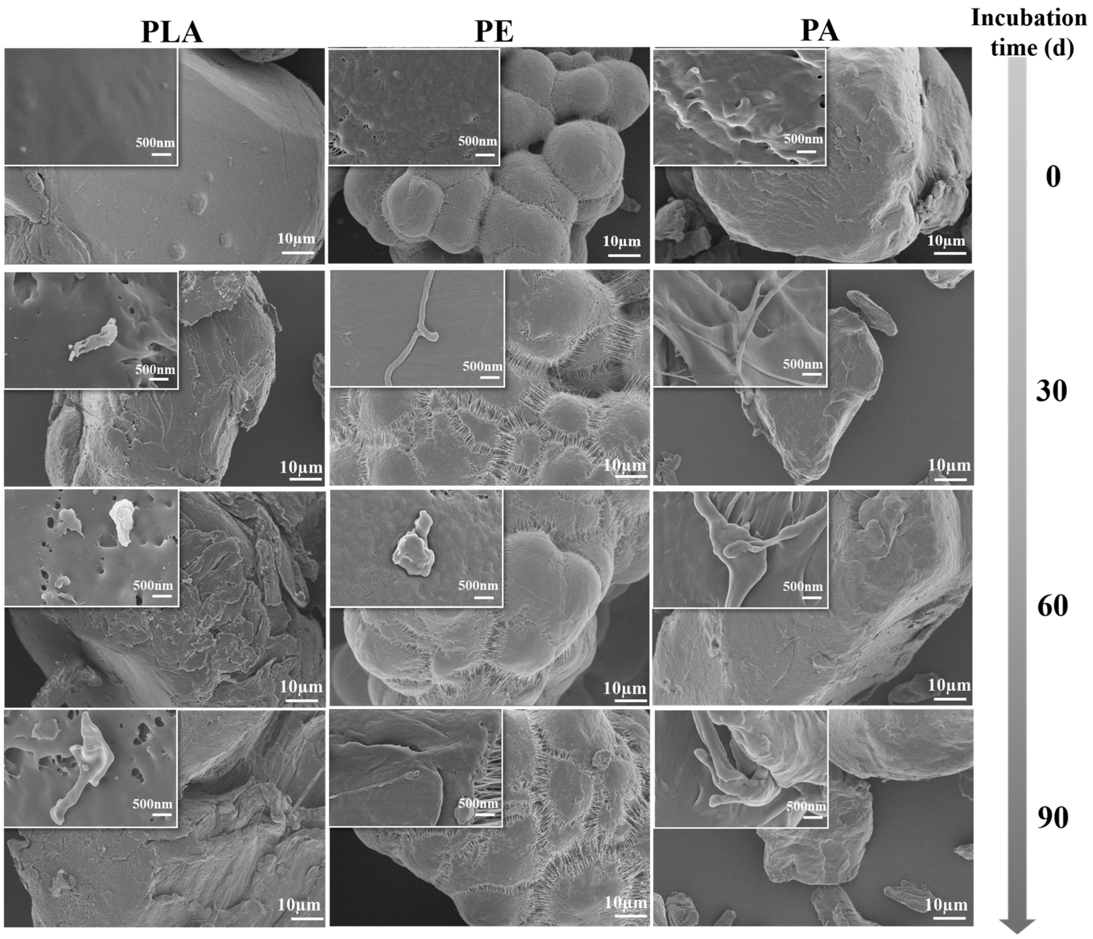

Figure S3. Field emission scanning electron microscopy images of three types of microplastics before and after incubation in yellow-brown soil.

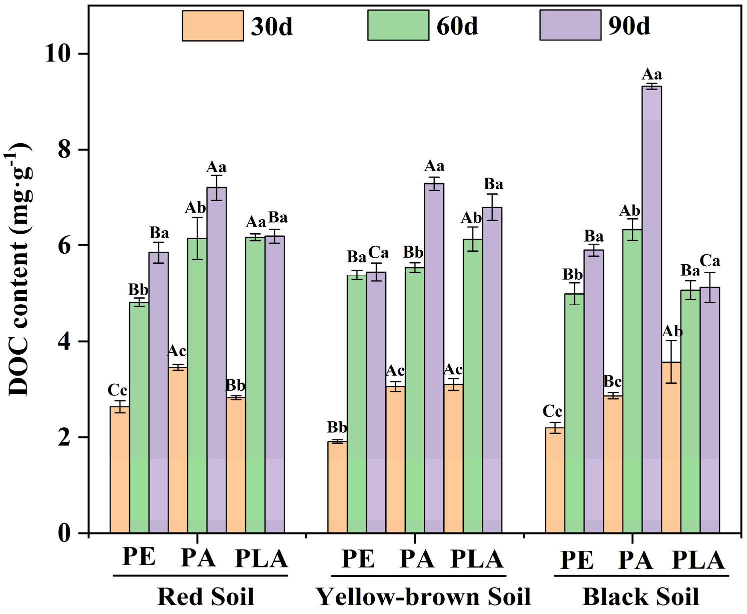

Figure S4. Temporal changes in the contents of dissolved organic carbon in EPS during incubation. The capital letters indicate significant differences of the contents of dissolved organic carbon in EPS from different MPs incubated in the same soil for same incubation duration, while the lowercase letters indicate significant differences of the contents of dissolved organic carbon in EPS from the same MPs incubated in the same soil across different incubation durations. The significance threshold was set at 0.05.

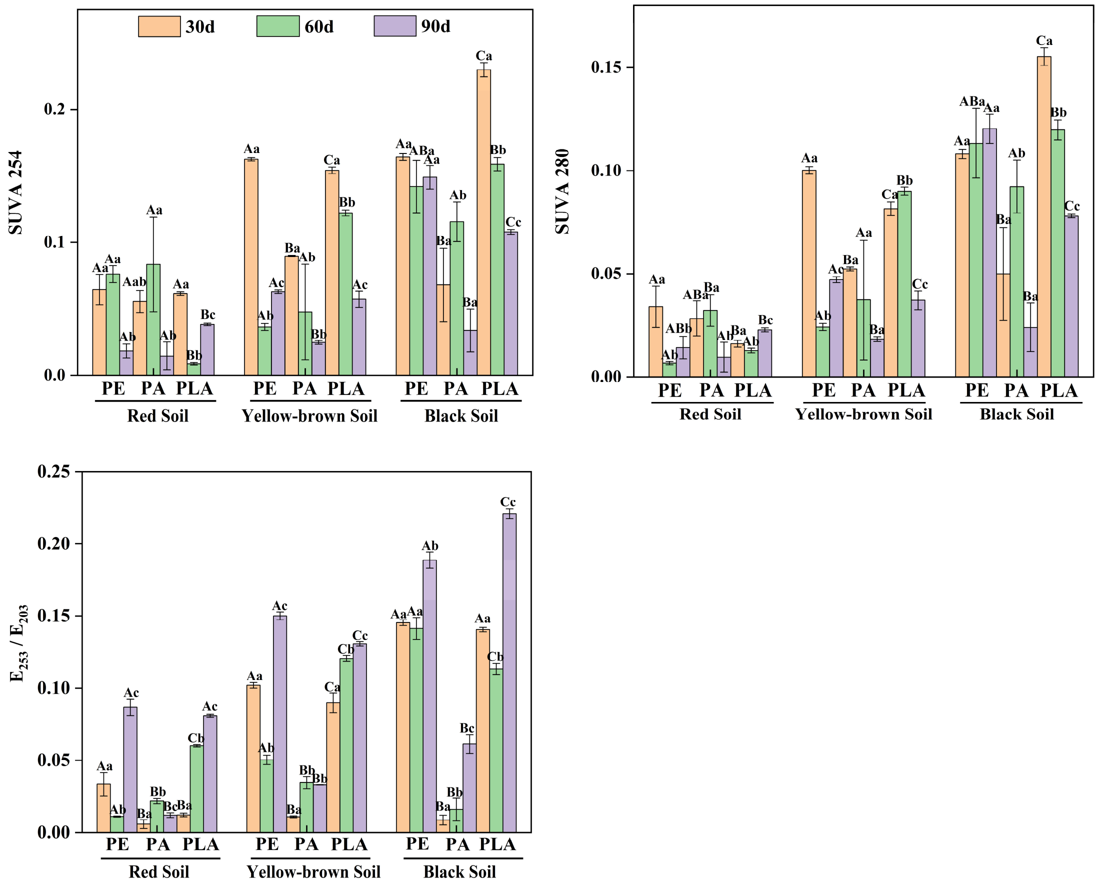

Figure S5. Temporal changes in UV-Vis spectral parameters of EPS, including SUVA254, SUVA 280 and E_{253}/E_{203}, during incubation. The capital letters indicate the significant differences of the spectral parameters of EPS from different MPs incubated in the same soil for same incubation duration, while the lowercase letters indicate significant differences in spectral parameters of EPS from the same MPs incubated in the same soil across different incubation durations. The significance threshold was set at 0.05.

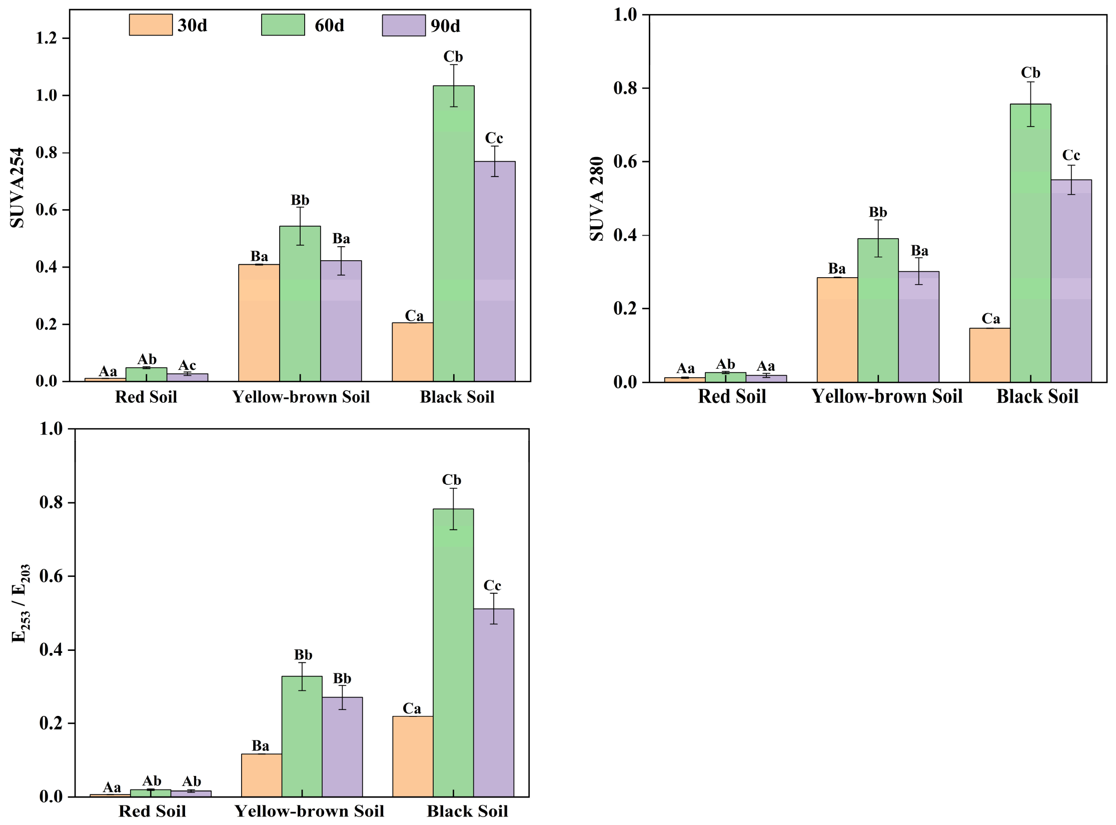

Figure S6. Temporal changes in UV-Vis spectral parameters of soil DOM, including SUVA254, SUVA 280 and E_{253}/E_{203}, during incubation. The capital letters indicate significant differences in the UV-Vis spectral parameters among different soil types at the same incubation time, while the lowercase letters indicate significant differences in UV-Vis spectral parameters across different incubation durations within same soil type. The significance threshold was set at 0.05.

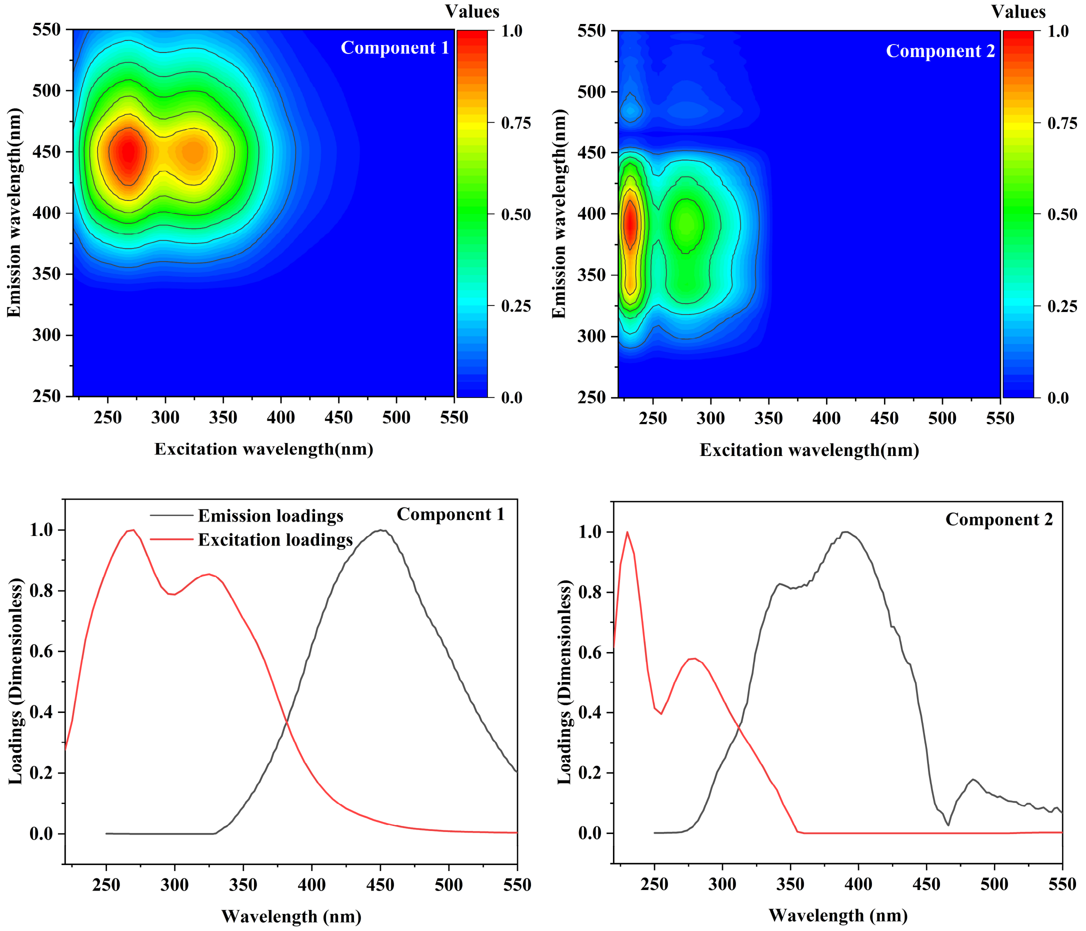

Figure S7. Two main components of soil DOM derived from parallel factor analysis modeling.

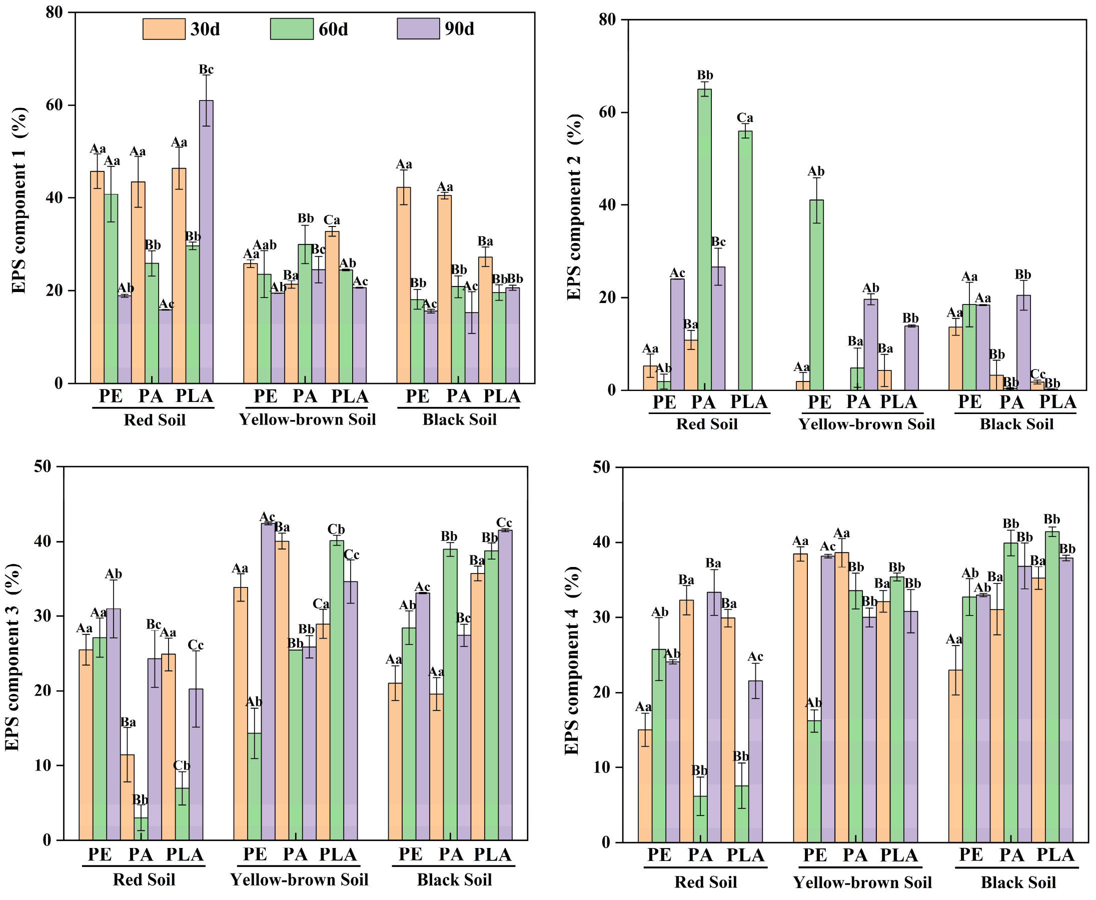

Figure S8. The changes of relative abundances of four main components of plastisphere EPS during incubation period. The capital letters indicate significant differences in the relative abundances of EPS components from different MPs incubated in the same soil for same incubation duration, while the lowercase letters indicate significant differences in the relative abundances in EPS components from the same MPs incubated in the same soil across different incubation durations. The significance threshold was set at 0.05.

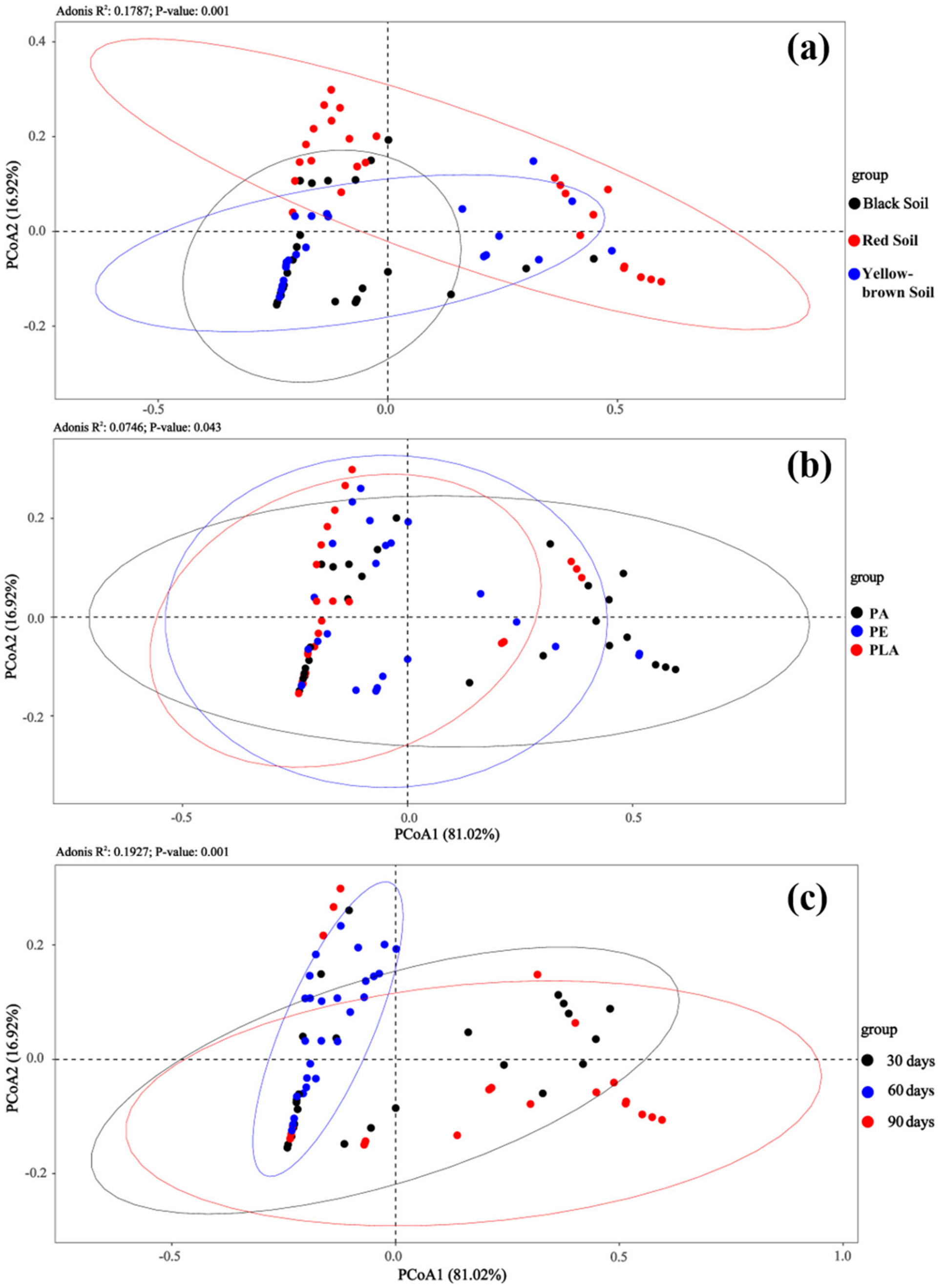

Figure S9. PEARMANOVA analysis was performed to measure the influence of colonization environment (a), polymer types (b) and incubation time (c) on the composition of plastisphere EPS.

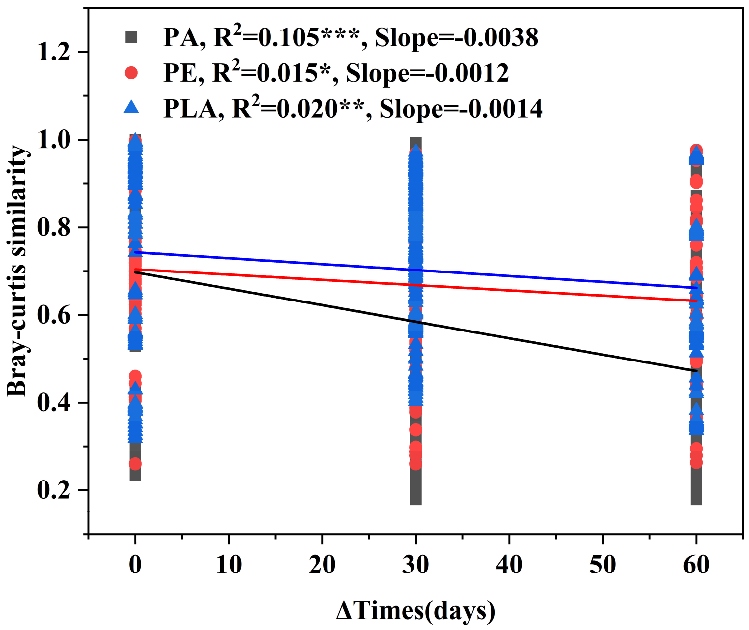

Figure S10. Response of similarity of plastisphere EPS composition to the temporal differences in incubation. * represent p <0.05, ** represent p <0.01, *** represent p <0.001.

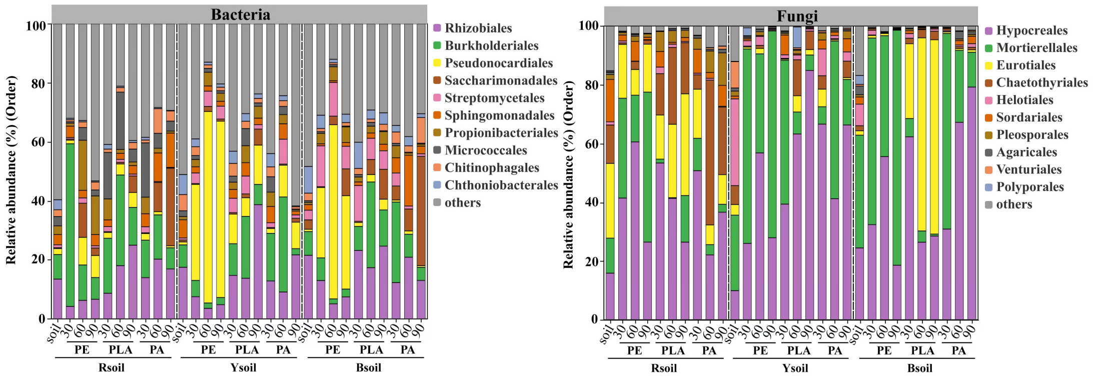

Figure S11. Heatmap of the abundance of the top 10 most abundant (a) bacterial and (b) fungal orders in the plastispheres during the whole incubation.

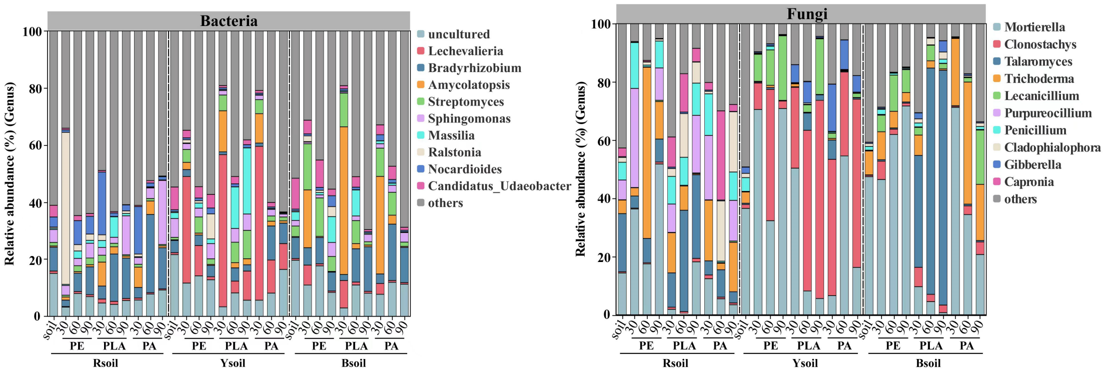

Figure S12. Heatmap of the abundance of the top 10 most abundant (a) bacterial and (b) fungal genera in the plastisphere during the entire incubation period.

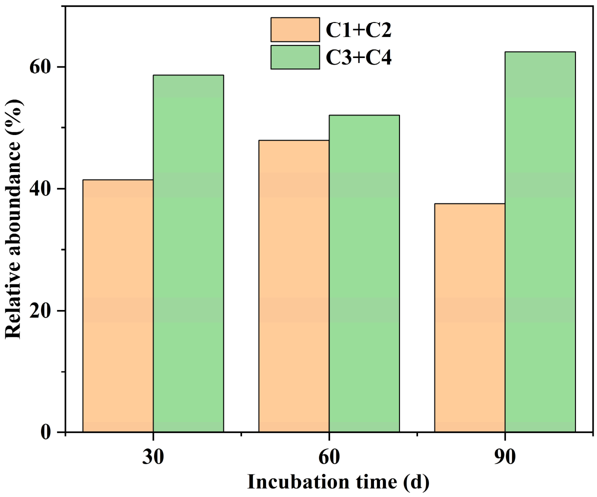

Figure S13. Relative abundance of the sums of EPS components 1+2 and components 3+4. The four main components of plastisphere extracellular polymeric substances, derived from parallel factor analysis, are designated C1 to C4.

Table S1. Mean and SD values of EPS components

Factor	C1(%)		C2(%)		C3(%)		C4(%)	
	Mean	S.D.	Mean	S.D.	Mean	S.D.	Mean	S.D.
RPE30	45.76	3.71	5.29	2.50	25.50	2.04	15.02	2.23
RPE60	40.79	6.04	1.88	1.60	27.11	2.63	25.76	4.21
RPE90	18.89	0.32	24.00	0.03	30.97	3.88	24.07	0.27
YPE30	25.82	0.84	1.91	1.91	33.83	1.83	38.44	0.93
YPE60	23.56	5.06	40.99	4.95	14.32	3.36	16.19	1.48
YPE90	19.45	0.04	0.00	0.00	42.38	0.19	38.17	0.23
BPE30	42.30	3.80	13.71	1.83	21.01	2.34	22.98	3.28
BPE60	18.08	2.14	18.55	4.77	28.44	2.23	32.71	2.46
BPE90	15.53	0.39	18.43	0.10	33.09	0.09	32.95	0.20
RPLA30	46.44	4.55	0.00	0.00	24.88	2.17	29.92	1.19
RPLA60	29.62	0.80	55.92	1.58	6.93	2.23	7.53	3.02
RPLA90	60.99	5.53	0.00	0.00	20.25	5.12	21.52	2.36
YPLA30	32.75	1.03	4.27	3.45	28.95	1.92	32.11	1.43
YPLA60	24.47	0.17	0.00	0.00	40.15	0.69	35.39	0.51
YPLA90	20.61	0.09	13.96	0.24	34.64	2.90	30.79	2.89
BPLA30	27.28	2.08	1.79	0.40	35.71	0.95	35.23	1.54
BPLA60	19.58	1.65	0.26	0.08	38.74	1.10	41.41	0.63
BPLA90	20.58	0.54	0.00	0.00	41.49	0.19	37.93	0.35
RPA30	43.45	5.49	10.84	2.07	11.46	3.63	32.29	1.97
RPA60	25.89	2.72	64.97	1.59	2.98	1.74	6.17	2.57
RPA90	15.82	0.07	26.59	4.02	24.28	3.82	33.31	3.05
YPA30	21.33	0.82	0.00	0.00	40.06	1.07	38.62	1.89
YPA60	29.92	4.10	4.90	4.24	25.47	0.00	33.53	2.36
YPA90	24.52	2.90	19.63	1.15	25.88	1.50	30.01	1.27
BPA30	40.48	0.74	3.24	3.24	19.57	2.19	31.09	3.44
BPA60	20.86	2.37	0.42	0.14	38.95	0.95	39.91	1.70
BPA90	15.22	4.52	20.49	3.22	27.44	1.49	36.85	3.08

Table S2. Mean and SD values of DOC and key EPS parameters

Factor	DOC ($mg \cdot g^{-1}$)		HIX		FI		BIX		Freshness index	
	Mean	S.D.	Mean	S.D.	Mean	S.D.	Mean	S.D.	Mean	S.D.
RPE30	2.63	0.12	0.21	0.15	1.81	0.10	0.76	0.01	1.30	0.05
RPE60	4.81	0.09	0.19	0.12	1.73	0.11	1.54	0.02	1.36	0.02
RPE90	5.85	0.22	0.38	0.08	1.87	0.01	0.97	0.01	0.89	0.04
YPE30	1.91	0.03	0.38	0.03	1.50	0.01	0.92	0.07	0.88	0.06
YPE60	5.39	0.10	0.42	0.06	1.42	0.03	0.94	0.04	0.90	0.04
YPE90	5.45	0.19	0.53	0.04	1.44	0.02	0.69	0.01	0.67	0.01
BPE30	2.20	0.12	0.54	0.01	1.40	0.06	0.76	0.01	0.74	0.01
BPE60	4.99	0.23	0.41	0.01	1.39	0.03	0.84	0.01	0.83	0.01
BPE90	5.90	0.12	0.57	0.02	1.40	0.01	0.69	0.01	0.68	0.01
RPLA30	2.82	0.05	0.29	0.06	1.72	0.03	1.06	0.03	0.98	0.04
RPLA60	6.17	0.07	0.20	0.13	1.73	0.05	1.29	0.06	1.14	0.07
RPLA90	6.19	0.15	0.17	0.07	1.78	0.03	0.93	0.03	0.87	0.02
YPLA30	3.10	0.12	0.55	0.05	1.37	0.01	0.73	0.02	0.72	0.02
YPLA60	6.13	0.25	0.36	0.01	1.37	0.00	0.95	0.03	0.89	0.03
YPLA90	6.79	0.27	0.51	0.02	1.42	0.02	0.64	0.02	0.63	0.01
BPLA30	3.57	0.44	0.55	0.06	1.39	0.03	0.78	0.06	0.76	0.05
BPLA60	5.07	0.19	0.45	0.02	1.45	0.07	0.85	0.02	0.81	0.02
BPLA90	5.13	0.32	0.56	0.02	1.41	0.01	0.67	0.01	0.66	0.00
RPA30	3.46	0.07	0.26	0.05	1.85	0.03	1.16	0.07	1.08	0.03
RPA60	6.14	0.44	0.19	0.03	1.79	0.03	1.18	0.05	1.10	0.05
RPA90	7.20	0.26	0.21	0.13	1.96	0.08	1.01	0.02	0.93	0.02
YPA30	3.06	0.10	0.39	0.00	1.54	0.01	0.90	0.03	0.87	0.03
YPA60	5.54	0.10	0.29	0.09	1.60	0.02	0.98	0.04	0.92	0.03
YPA90	7.28	0.14	0.32	0.05	1.89	0.03	1.04	0.11	0.95	0.09
BPA30	2.86	0.07	0.54	0.03	1.50	0.01	1.04	0.11	0.83	0.01
BPA60	6.33	0.22	0.30	0.10	1.73	0.14	1.10	0.17	1.02	0.14
BPA90	9.32	0.06	0.44	0.19	1.84	0.04	0.89	0.05	0.85	0.05

Table S3. The accumulation rate of dissolved organic carbon (DOC) in EPS

Soil type	Plastic type	Change rate of DOC in EPS (mg C g^{-1} MP day^{-1})		
		0-30 day	30-60 day	60-90day
Red soil	PE	0.088	0.073	0.035
	PA	0.115	0.090	0.035
	PLA	0.094	0.112	0.001
Yellow-brown soil	PE	0.064	0.116	0.002
	PA	0.102	0.083	0.058
	PLA	0.103	0.101	0.022
Black soil	PE	0.073	0.093	0.030
	PA	0.095	0.115	0.100
	PLA	0.119	0.050	0.002

Table S4. Results of Tucker's Congruency Coefficients (TCC) for soil dissolved organic matter. Commonly, TCC > 0.95 indicates a good similarity.

	Component	Combination	TCC-em	TCC-ex
1	Comp.1	AB vs CD	0.9979221	0.9983581
2	Comp.1	AC vs BD	0.9993651	0.9998644
3	Comp.1	AD vs BC	0.9989367	0.9997106
4	Comp.2	AB vs CD	0.9822341	0.9533271
5	Comp.2	AC vs BD	0.9962260	0.9908517
6	Comp.2	AD vs BC	0.9898189	0.9741305

Table S5. Results of Tucker's Congruency Coefficients (TCC) for plastisphere extracellular polymeric substances. Commonly, TCC > 0.95 indicates a good similarity.

	Component	Combination	TCC-em	TCC-ex
1	Comp.1	AB vs CD	0.9970021	0.9885619
2	Comp.1	AC vs BD	0.9986643	0.9974075
3	Comp.1	AD vs BC	0.9943404	0.9984630
4	Comp.2	AB vs CD	0.9939624	0.9983441
5	Comp.2	AC vs BD	0.9976549	0.9972973
6	Comp.2	AD vs BC	0.9973831	0.9986193
7	Comp.3	AB vs CD	0.9853717	0.9762880
8	Comp.3	AC vs BD	0.9909886	0.9917817
9	Comp.3	AD vs BC	0.9922609	0.9918893
10	Comp.4	AB vs CD	0.9977920	0.9978364
11	Comp.4	AC vs BD	0.9960292	0.9927557
12	Comp.4	AD vs BC	0.9901758	0.9898211

Table S6. Spearman correlation coefficients between top 10 bacterial and fungal genera and EPS components.

	Genera	Component1	Component2	Component3	Component4
		r	r	r	r
Bacterial genera	uncultured	-0.40*	0.07	0.10	0.10
	Lechevalieria	-0.09	-0.08	0.29	0.18
	Bradyrhizobium	0.10	0.02	-0.33	-0.14
	Amycolatopsis	0.01	-0.09	0.15	-0.03
	Streptomyces	-0.32	-0.29	0.65***	0.52**
	Sphingomonas	0.20	-0.02	-0.17	-0.08
	Massilia	0.36	-0.37	0.37	0.34
	Ralstonia	0.16	0.05	-0.06	-0.14
	Nocardioides	0.15	0.05	-0.10	-0.15
	Candidatus_Udaeobacter	-0.25	-0.26	0.57**	0.39*
Fungal genera	Mortierella	-0.26	-0.06	0.25	0.20
	Clonostachys	-0.21	-0.04	0.22	0.22
	Talaromyces	0.37	-0.06	-0.17	-0.13
	Trichoderma	-0.02	0.31	-0.31	-0.23
	Lecanicillium	-0.45*	0.08	0.16	0.15
	Purpureocillium	-0.24	0.42*	-0.48*	-0.50**
	Penicillium	0.12	0.20	-0.42*	-0.40*
	Cladophialophora	0.17	0.17	-0.45*	-0.31
	Gibberella	0.05	-0.26	0.32	0.36
	Capronia	0.39*	0.15	-0.44*	-0.27

Table S7. Bacterial OTUs serving as nodes in the networks between components of plastisphere extracellular polymeric substances and bacterial community. The four main components of plastisphere extracellular polymeric substances, derived from parallel factor analysis modeling, were represented by C1 to C4.

Incubation durations (d)	Components	Bacterial OTUs	Total bacterial OTUs
30	C1	180	3442
	C2	1364	
	C3	960	
	C4	1670	
60	C1	1503	3024
	C2	31	
	C3	1294	
	C4	413	
90	C1	361	1596
	C2	133	
	C3	1000	
	C4	539	

Table S8. Fungal OTUs serving as nodes in the networks between components of plastisphere extracellular polymeric substances and fungal community. The four main components of plastisphere extracellular polymeric substances, derived from parallel factor analysis modeling, were represented by C1 to C4.

Incubation durations (d)	Components	Fungal OTU	Total fungal OTU
30	C1	19	234
	C2	93	
	C3	49	
	C4	98	
60	C1	85	175
	C2	15	
	C3	42	
	C4	40	
90	C1	32	68
	C2	18	
	C3	9	
	C4	13	

REFERENCES

1. Chen, M. L.; Liu, S. S.; Bi, M. H.; Yang, X. Y.; Deng, R. Y.; Chen, Y. Aging behavior of microplastics affected DOM in riparian sediments: From the characteristics to bioavailability. *J. Hazard Mater.* **2022**, 431, 128522.
2. Deng, W. B.; Wang, Y. J.; Wang, Z. H.; Liu, J. X.; Wang, J.; Liu, W. J. Effects of photoaging on structure and characteristics of biofilms on microplastic in soil: Biomass and microbial community. *J. Hazard. Mater.* **2024**, 467, 133726.
3. Murphy, K. R.; Stedmon, C. A.; Graeber, D.; Bro, R. Fluorescence spectroscopy and multi-way techniques. PARAFAC. *Anal. Methods.* **2013**, 5, 6557-6566.
4. Zhang, Y. Z.; Liu, Y. D.; Zhou, A. G.; Zhang, L. Identification of groundwater pollution from livestock farming using fluorescence spectroscopy coupled with multivariate statistical methods. *Water Res.* **2021**, 206, 117754.
5. Chen, R. Q.; Chen, T. Y.; Zhou, Y. K.; Li, L. F.; Li, L. X. Y.; Zhu, N. L.; Li, Z. G.; Wang, Y. F.; Jiang, G. B. Characteristics of disinfection byproducts from dissolved organic matter during chlor(am)ination of source water in Tibetan Plateau, China. *Sci. Total Environ.* **2024**, 947, 174628.
6. Sun, Y. Z.; Li, X. F.; Li, X. M.; Wang, J. Deciphering the fingerprint of

dissolved organic matter in the soil amended with biodegradable and conventional microplastics based on optical and molecular signatures. *Environ. Sci. Technol.* **2022**, 56 (22), 15746-15759.

7. Yu, S. Y.; Lv, J. T.; Jiang, L.; Geng, P. Y; Cao, D.; Wang, Y. W. Changes of soil dissolved organic matter and its relationship with microbial community along the Hailuogou glacier forefield chronosequence. *Environ. Sci. Technol.* **2023**, 57 (9), 4027-4038.
